# Nitric Oxide Is an Activity-Dependent Regulator of Target Neuron Intrinsic Excitability

**DOI:** 10.1016/j.neuron.2011.05.037

**Published:** 2011-07-28

**Authors:** Joern R. Steinert, Susan W. Robinson, Huaxia Tong, Martin D. Haustein, Cornelia Kopp-Scheinpflug, Ian D. Forsythe

**Affiliations:** 1 Neurotoxicity at the Synaptic Interface, MRC Toxicology Unit, Hodgkin Building, University of Leicester, Leicester LE1 9HN, UK

## Abstract

Activity-dependent changes in synaptic strength are well established as mediating long-term plasticity underlying learning and memory, but modulation of target neuron excitability could complement changes in synaptic strength and regulate network activity. It is thought that homeostatic mechanisms match intrinsic excitability to the incoming synaptic drive, but evidence for involvement of voltage-gated conductances is sparse. Here, we show that glutamatergic synaptic activity modulates target neuron excitability and switches the basis of action potential repolarization from Kv3 to Kv2 potassium channel dominance, thereby adjusting neuronal signaling between low and high activity states, respectively. This nitric oxide-mediated signaling dramatically increases Kv2 currents in both the auditory brain stem and hippocampus (>3-fold) transforming synaptic integration and information transmission but with only modest changes in action potential waveform. We conclude that nitric oxide is a homeostatic regulator, tuning neuronal excitability to the recent history of excitatory synaptic inputs over intervals of minutes to hours.

## Highlights

► Synaptic input drives NO-mediated modulation of voltage-gated potassium currents ► High synaptic activity switches the dominant delayed rectifier to Kv2 ► NO volume transmission tunes target neurons to excitatory synaptic drive ► This homeostatic regulation occurs broadly in the brain (brain stem and hippocampus)

## Introduction

A multitude of control mechanisms act to tune ion channel activity to neuronal function and network activity, thereby refining synaptic integration and the computation encoded in the action potential (AP) output (Marder and Goaillard, 2006; Nelson and Turrigiano, 2008). Activity-dependent processes are clearly associated with synaptic scaling and long-term changes in synaptic strength that enhance or suppress the ability of particular synaptic inputs to trigger postsynaptic APs, with many of these mechanisms (such as LTP and LTD) underlying learning and memory (Morris et al., 2003). Many studies show changes in synaptic strength, but synaptic activity can also regulate voltage-gated conductances (Frick et al., 2004). We postulate that nitrergic signaling links synaptic activity to the control of postsynaptic intrinsic excitability in many areas of the brain, including the hippocampus (Frick et al., 2004; Misonou et al., 2004; Mohapatra et al., 2009; van Welie et al., 2006) and auditory brain stem (Song et al., 2005; Steinert et al., 2008).

Neuronal excitability is determined by the expression, location, and activity of voltage-gated ion channels in the plasma membrane. Na^+^ and Ca^2+^ channels dominate AP generation, but the crucial regulators of excitability are voltage-gated potassium (K^+^) channels. There are over 40 α subunit K^+^ channel genes (Coetzee et al., 1999; Gutman et al., 2003) associated with 12 families (Kv1–12). A native channel requires four α subunits (usually from within the same family) with heterogeneity providing a spectrum of channel kinetics. They set resting membrane potentials, neuronal excitability, AP waveform, firing threshold, and firing rates. Here, we focus on two broadly expressed families: Kv2 (Du et al., 2000; Guan et al., 2007; Johnston et al., 2008), and Kv3 (Rudy et al., 1999; Rudy and McBain, 2001; Wang et al., 1998), which are well characterized and underlie many neuronal “delayed rectifiers” (Hodgkin and Huxley, 1952) throughout the nervous system.

Both Kv2 and Kv3 are “high voltage-activated channels (HVAs),” requiring depolarization to the relatively positive voltages achieved during an AP, with half-activation voltages around 0 mV (±20 mV, dependent on subunit composition, accessory subunits, and phosphorylation). Kv2 channels have a broader activation range and slower kinetics than Kv3, so that Kv2 starts to activate close to AP threshold and is slower to deactivate (and slower to inactivate). The subcellular localization of Kv2 and Kv3 channels differs substantially; Kv2 channels are often clustered or “corralled” (Misonou et al., 2004; Muennich and Fyffe, 2004; O'Connell et al., 2006) and are localized to axon initial segments (AISs) (Johnston et al., 2008; Sarmiere et al., 2008) or proximal dendrites. Kv3.1 channels can be found in postsynaptic soma and AIS and are sometimes located at nodes of Ranvier (Devaux et al., 2003) and on the nonrelease face of excitatory synapses (Elezgarai et al., 2003). Distinction between native Kv3 and Kv2 channels is best based on their pharmacology: Kv3 channels are blocked by low concentrations (1 mM) of tetraethylammonium (TEA) (Grissmer et al., 1994; Wang et al., 1998), whereas Kv2 channel gating is shifted to more positive voltages by r-stromatoxin-1 (Escoubas et al., 2002). Most neuronal Kv2 channels contain Kv2.1 subunits, as in the hippocampus (Du et al., 2000), whereas Kv2.2 has a more restricted expression, such as the medial nucleus of the trapezoid body (MNTB) (Johnston et al., 2008).

Neuronal nitric oxide synthase (nNOS) is widely expressed in the brain, activated by Ca^2+^ influx through synaptic NMDARs (Brenman et al., 1996; Garthwaite et al., 1988) and linked with synaptic plasticity in the cerebellum (Boxall and Garthwaite, 1996; Shin and Linden, 2005), hippocampus (Lu et al., 1999), and neocortex (Hardingham and Fox, 2006). Nitric oxide (NO) is associated with signaling across many physiological systems, including cardiovascular, immune, and enteric and central nervous systems, and related to disease and pathological states (Garthwaite, 2008; Steinert et al., 2010a). nNOS is often localized to subpopulations of neurons in a given region, and the source or the specific targets of nitrergic signaling are hard to identify at a molecular level or in a physiological context. Soluble guanylyl cyclase (sGC) is the major NO receptor and hence, cGMP-mediated activation of PKG and subsequent changes in the balance of kinase/phosphatase activity modulates target protein phosphorylation, such as ligand- (Serulle et al., 2007) and voltage-gated ion channels (Park et al., 2006). Recent evidence from the auditory brain stem demonstrates that Kv3.1 channels are a target for cGMP/NO-signaling pathways following synaptic activity (Steinert et al., 2008). NO is also postulated to act as a retrograde transmitter, and although presynaptic actions are known (Garthwaite, 2008), i.e., through volume transmission (Artinian et al., 2010; Steinert et al., 2008), the present study focuses on signaling to postsynaptic targets.

Expression of Kv3 and Kv2 channels in association with NO and glutamatergic signaling occurs broadly in the brain, including the auditory brain stem (Johnston et al., 2008; Steinert et al., 2008) and hippocampus (Tansey et al., 2002). In this study nitrergic signaling was activated by sustained excitatory synaptic activity (10 Hz) for around 1 hr, modulating excitability of principal neurons in the MNTB and CA3 pyramidal neurons by suppression of Kv3 conductances and dramatic enhancement of Kv2 currents. This switched the drive for AP repolarization to Kv2 channels, raising firing threshold and altering AP responses in both brain regions. The nitrergic facilitation of Kv2 implies that this conductance is more dominant in vivo than previously suspected because recording within minutes of animal sacrifice shows vastly enhanced Kv2 currents.

## Results

Throughout this study, we used whole-cell patch recording in an unpaired fashion: control data were recorded from one population of neurons, then conditioning stimulation was applied to the synaptic pathways, followed by test recordings from another population of neurons; this avoided dialysis inherent in long-term recording from the same neuron. The objective was to test if sustained excitatory synaptic input to a target neuron changed its intrinsic excitability. This is distinct from short-term depression of synaptic responses observed following short periods of conditioning spontaneous activity (Hennig et al., 2008; Hermann et al., 2007) in that our studies focused on how sustained synaptic inputs can influence postsynaptic voltage-gated conductances rather than synaptic strength. The conditioning synaptic stimulation lasted 1 hr and consisted of evoked EPSPs at a mean frequency of 10 Hz (with interstimulus intervals [ISIs] generated by a Poisson process, giving a total of 34,875 stimuli/1 hr). We stimulated the trapezoid body calyceal projection to the MNTB or mossy fiber/commissural projections (which were DCG-IV insensitive; see Figure S1C available online) to CA3 pyramidal neurons. Stimulation at 10 Hz induces neither LTP nor LTD (Dudek and Bear, 1992) and provided a sustainable stimulation rate that did not deplete transmission to subthreshold levels (Figure S1A, stimulus recordings at 55 min) and was comparable with physiological firing rates for the MNTB (Kopp-Scheinpflug et al., 2003) and hippocampus (Fenton and Muller, 1998; Klyachko and Stevens, 2006).

### Sustained Synaptic Activity Increased Transmission Fidelity and Reduced Neuronal Excitability

**Figure 1:**
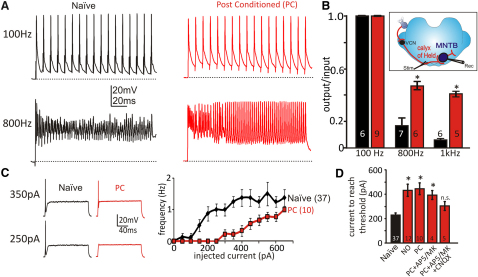
Synaptic Conditioning Enhances High-Frequency Firing and Reduces Postsynaptic Excitability in MNTB Neurons (A) Left view illustrates synaptically evoked 100 Hz (upper) and 800 Hz (lower) trains delivered before conditioning (Naive, black) showed failures after 4–6 APs at 800 Hz. Right view shows 100 and 800 Hz trains after synaptic conditioning at 10 Hz for 1 hr with Poisson-distributed ISIs (PC, red); note improved AP firing maintained through the 800 Hz train in contrast to Naive (black). (B) Summary of MNTB output/input ratios: Naive (black) and PC (red), PC resulted in improved fidelity at high frequencies (100 Hz: Naive 1 ± 0, PC 1 ± 0; 800 Hz: Naive 1.7 ± 0.8, PC 0.5 ± 0.4, p = 0.0089; 1 kHz: naive 0.06 ± 0.01, PC 0.4 ± 0.2, p < 0.0001). Inset shows orientation of slice, synaptic input, and stimulation (Stim) and recording (Rec) electrodes. (C) Left view is example traces for APs evoked by current injection in Naive (black), showing reduced excitability after PC (red). Right view is a frequency plot of current evoked APs; PC reduced postsynaptic excitability. (D) AP firing threshold is increased by synaptic conditioning or nitrergic signaling: Naive, control; NO, following NO-donor incubation (1 hr, 100 μM SNP or PapaNONOate); PC, synaptic conditioning. The threshold rise is suppressed by blocking glutamate receptors: PC+AP5/MK+CNQX (50 μM AP5 + 10 μM MK801 + 10 μM CNQX). Statistical significance was determined by one-way ANOVA with posttest; data tested versus Naive. Error bars are mean ± SEM. ^∗^p < 0.01, n.s., not significant, unpaired data.

In naive slices under current clamp recording, evoked EPSP trains at moderate frequencies securely triggered APs in principal neurons of the MNTB (<400 Hz). The illustrated example in Figure 1 shows single AP responses to each presynaptic stimulus at a frequency of 100 Hz (Figure 1A, Naive, upper black). But transmission failure occurred rapidly at 800 Hz or above (Figure 1A, Naive, lower black), consistent with previous reports (Taschenberger and von Gersdorff, 2000). After synaptic conditioning (post-conditioning, PC: 1 hr stimuli), the response of MNTB neurons to moderate frequency stimuli was robust and unchanged (Figure 1A, upper red trace; 100 Hz, PC), but high-frequency stimuli now triggered APs with greater reliability (Figure 1A, PC, lower red trace; 800 Hz). The conditioning reduced evoked synaptic currents (Figure S1B), consistent with nitrergic suppression of AMPARs reported previously (Steinert et al., 2008).

Comparison of the mean output (MNTB APs) to input (at 100, 800, or 1000 Hz) for naive (Figure 1B, black bars) and PC slices (red bars) showed increased reliability of transmission for high-frequency stimulation after conditioning. The synaptic conditioning also increased AP threshold (Figure 1C), consistent with reduced postsynaptic excitability. AMPAR and NMDAR antagonists (50 μM AP-5, 10 μM MK801, 10 μM CNQX applied for the 1 hr conditioning period) blocked these changes, whereas perfusion of NO donors (NO: sodium nitroprusside, SNP or PapaNONOate, each 100 μM for 1 hr) mimicked the threshold increase (Figure 1D).

**Figure 2:**
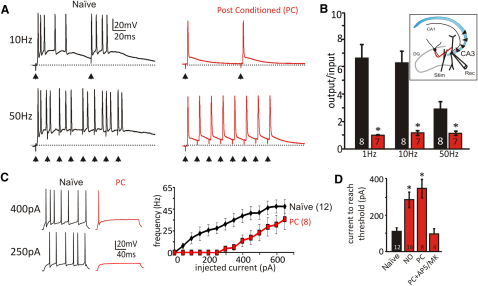
Synaptic Conditioning Enhances Transmission Fidelity and Reduces Excitability of CA3 Pyramidal Neurons (A) Synaptically evoked 10 and 50 Hz trains delivered before conditioning (Naive, black) show multiple spiking to each evoked EPSP (stimulus indicated by black arrowhead). Right view shows that after synaptic stimulation conditioning at 10 Hz for 1 hr with Poisson-distributed ISIs (PC, red), only single APs are triggered by each evoked EPSP (PC, red). (B) CA3 output/input ratios show that PC (red bar) reduced the number of APs to each evoked EPSP (indicated by black arrowheads), so the CA3 output/input ratios were 1:1 for frequencies from 1–50 Hz; p < 0.01 (one-way ANOVA with posttest). Inset shows stimulation (Stim) and recording (Rec) sites within the hippocampal slice. (C) Left view is example traces showing APs evoked by current injection from Naive (black) neurons, showing reduced excitability from CA3 neurons after synaptic conditioning (PC, red). Right view is a frequency plot of current evoked APs; PC reduced postsynaptic excitability, so fewer APs were evoked for a given injected current. (D) AP firing threshold is increased by synaptic conditioning or nitrergic signaling: Naive, control; NO, following NO-donor incubation (1 hr, 100 μM SNP or PapaNONOate); PC, after synaptic conditioning. The threshold rise is suppressed by blocking NMDARs: PC+AP5/MK (50 μM AP5 + 10 μM MK801). Statistical significance was determined by one-way ANOVA with posttest; data tested versus Naive. Error bars are mean ± SEM. ^∗^p < 0.05, n.s., not significant, unpaired data.

Analogous changes in excitability also occurred in the hippocampus, where naive CA3 pyramidal neurons (CA3 neurons) responded with AP bursts to stimulation of the mossy fiber/commissural synaptic inputs (Brown and Randall, 2009) across the range of 10–50 Hz (Shao and Dudek, 2009) (Figure 2A, Naive, black). The same conditioning paradigm reduced CA3 neuron excitability so that each EPSP triggered a maximum of one AP (Figure 2A, PC, red), and output/input ratios became close to 1:1 (Figure 2B). The conditioning induced no change in evoked synaptic currents (Figure S1C), demonstrating that this 10 Hz stimulation paradigm did not significantly influence synaptic strength (Dudek and Bear, 1992).

The postsynaptic locus of this excitability change was again confirmed by testing excitability with injection of current steps: naive CA3 neurons fired multiple APs, increasing in numbers proportionally with depolarizing current injection (Figure 2C, Naive, black), but following synaptic conditioning, the current threshold for AP generation was raised (Figure 2C, PC, red) from 100 to over 300 pA. The PC-induced threshold rise was blocked by NMDAR antagonists (50 μM AP-5, 10 μM MK801 applied for the 1 hr conditioning) and mimicked by perfusion of NO donors (Figure 2D), consistent with a nitrergic decrease in excitability.

These results gave two general insights into the control of neuronal excitability: glutamatergic synaptic activity reduced excitability of the target neurons in the MNTB and CA3, and this was mediated by NO signaling. We next explored the mechanism of this postsynaptic excitability change using whole-cell voltage clamp.

### NO Signaling Markedly Potentiates Outward K^+^ Currents in MNTB and CA3 Pyramidal Neurons

**Figure 3:**
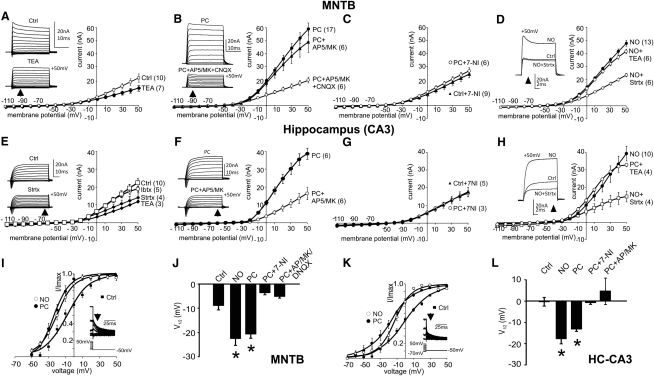
NO Triggers a Transition in the K^+^ Channels Mediating AP Repolarization in Brain Stem and CA3 Pyramidal Neurons MNTB data are shown in (A)–(D); CA3 data are shown in (E)–(H): average current-voltage (I/V) relationships are plotted for each condition as the mean of absolute current amplitudes and without leak subtraction. The data are unpaired (see Experimental Procedures; n is given by the numeral in brackets after conditions), and so each I/V curve is directly comparable to others in adjacent I/V plots. (A) I/V relationship of outward K^+^ currents for control (Ctrl) neurons is reduced in the presence of 1 mM TEA. Insets show current traces (command steps from −110 to +50 mV). (B) After synaptic conditioning (PC) outward currents are enhanced. This facilitation is blocked by combined NMDAR and AMPAR antagonism. Insets show current traces (command steps from −110 to +50 mV). (C) K^+^ current potentiation was also blocked by nNOS inhibition: in the presence of 7-NI (10 μM), I/V relationships for control (Ctrl+7-NI) and after synaptic conditioning (PC+7-NI) are of the same amplitude. (D) NO donors mimic the potentiation by synaptic conditioning: I/V relationship shows enhanced currents in the presence of NO donors, and this was unchanged in the presence of the Kv3 antagonist TEA (1 mM). The potentiated current is blocked by the Kv2-gating modifier r-stromatoxin-1 (Strtx; 300 nM). Insets show current traces at +50 mV for each condition. (E) I/V relationships for outward K^+^ currents for control (Ctrl) and in the presence of TEA (1 mM), Strtx (300 nM), or Ibtx (100 nM) indicating contributions from Kv3, Kv2, and BK channels under control conditions. Insets show current traces (command steps from −110 to +50 mV). (F) The outward K^+^ currents are vastly enhanced following synaptic conditioning (PC), and this potentiation is blocked by NMDAR antagonists (50 μM AP-5 + 10 μM MK801). Insets show current trace examples. (G) K^+^ current potentiation was also blocked by nNOS inhibition, with I/V relationships for control (Ctrl) and PC being identical in the presence of 7-NI (10 μM). (H) NO donors also potentiate outward K^+^ currents; this current is insensitive to TEA (1 mM) and so not mediated by Kv3 (or BK). Potentiation was suppressed by the Kv2-gating modifier, Strtx (300 nM), consistent with increased Kv2 conductance. Insets show current traces at +50 mV (Figure S2 for voltage protocol). Activation curves for the outward K^+^ currents were plotted for both the MNTB (I and J) and CA3 pyramidal neurons (K and L). (I) MNTB activation curves for control (filled square), following PC (filled circle) and NO donor (open circle). Boltzmann functions were fit to the magnitude of tail currents (inset). (J) MNTB: V_1/2_ for the conditions indicated, note that PC and NO donors induced a leftward shift of V_1/2_ (n as indicated in I/Vs). (K) CA3 activation curves for control (filled square), following PC (filled circle) and NO donor (open circle). Boltzmann functions were fit to the tail currents (inset). (L) CA3: V_1/2_ for the conditions indicated, note that PC and NO induced a leftward shift of V_1/2_ (n as indicated in I/Vs). ^∗^p < 0.05, one-way ANOVA with posttest. Data are denoted as mean ± SEM.

Under voltage clamp, MNTB neurons exhibited a mean outward current of 23 ± 1 nA at +50 mV (Figure 3A, Ctrl, n = 10), of which one-third was blocked by the Kv3 antagonist TEA (1 mM), confirming Kv3 contribution (Figure 3A, TEA, n = 7) and consistent with previous reports (Macica and Kaczmarek, 2001). Following synaptic conditioning, the outward current increased to 59 ± 4 nA (Figure 3B, PC, n = 17; p < 0.0001, unpaired data). This large increase in conductance was blocked by antagonism of both NMDARs and AMPARs during the conditioning period (Figure 3B, PC+AP5/MK+CNQX, n = 6).

Likewise, voltage clamp of CA3 neurons showed control outward currents of 21 ± 2 nA (Figure 3E, n = 10, at +50 mV) that increased to 38 ± 2 nA after conditioning (Figure 3F, n = 6; p = 0.0005, unpaired data). NMDAR inhibition during conditioning also blocked the K^+^ current potentiation in the CA3 neurons (Figure 3F, n = 6). In both the MNTB and CA3 neurons, inhibition of nNOS by 7-nitroindazole (7-NI, 10 μM) during conditioning also blocked the K^+^ current potentiation (Figures 3C and 3G).

Under control naive conditions, CA3 HVA currents of 21 ± 2 nA were sensitive to 1 mM TEA (Figure 3E, 12 ± 1 nA, 43% reduction at +50 mV). Iberiotoxin (Ibtx; 100 nM) block of BK channels caused only minor suppression of whole-cell currents (Figure 3E, 18 ± 1 nA, 14% reduction at +50 mV), whereas subsequent application of 1 mM TEA substantially reduced outward currents to 11 ± 1 nA (not shown, n = 5), consistent with Kv3 contributing approximately 29% of total outward current. The Kv2-gating modifier r-stromatoxin-1 (300 nM) (Escoubas et al., 2002; Guan et al., 2007) reduced outward control currents by 34% (Figure 3E, 14 ± 3 nA at +50 mV), consistent with Kv2 (Du et al., 2000; Mohapatra et al., 2009; Sarmiere et al., 2008) and Kv3 contributions to outward currents in CA3 neurons. Note that Ibtx only blocked outward currents at voltages greater than +40 mV, suggesting that BK does not contribute to single APs, which peak at around +20 mV, whereas TEA reduced outward currents at voltages greater than −10 mV (Figure 3E), thereby contributing to AP waveform shaping.

### Kv2 K^+^ Currents Are Potentiated by NO Signaling

Two different NO donors (SNP or PapaNONOate, each 100 μM, 1 hr exposure, in the absence of synaptic stimulation) mimicked the synaptic conditioning and induced similarly large K^+^ currents in both MNTB and CA3 neurons (Figures 3D and 3H, NO), suggesting an activity-driven nitrergic modulation of currents. In contrast to control, these potentiated K^+^ currents were now insensitive to the Kv3 antagonist TEA (1 mM) (Brew and Forsythe, 1995; Grissmer et al., 1994) (Figures 3D and 3H, NO+TEA, PC+TEA), indicating that Kv3 does not contribute to outward K^+^ currents after nitrergic activation and consistent with suppression of Kv3 by NO in the MNTB (Steinert et al., 2008) or recombinant Kv3 channels expressed in CHO cells (Moreno et al., 2001). Clearly another voltage-activated K^+^ conductance was being potentiated in both MNTB and CA3, and this current was now largely suppressed by the Kv2-gating modifier r-stromatoxin-1 (Figures 3D and 3H, NO+Strtx, 300 nM, Figure S2), which acts on both Kv2.1 and Kv2.2 subunits (Escoubas et al., 2002; Guan et al., 2007). Kv2.1 is widely expressed in the hippocampus and cortical regions (Du et al., 2000; Mohapatra et al., 2009; Sarmiere et al., 2008), whereas Kv2.2 is highly expressed in the MNTB (Johnston et al., 2008), as supported by in situ hybridization by the Allen Brain Atlas (Lein et al., 2007). Kv2.2 was not detected in the CA3 region, and Kv2.1 was absent from MNTB principal cells (unpublished data).

Kv2 currents activate at potentials close to AP firing threshold (V_thr_) following conditioning (PC) in both the brain stem and hippocampus (MNTB V_thr_: −35 ± 2 mV, n = 10; CA3 V_thr_: −39 ± 2 mV, n = 8) and thereby set firing thresholds and excitability. The large modulation of the HVA K^+^ current has implications for AP firing threshold. Analysis of MNTB currents at −30 mV (around AP threshold) revealed a significant increase (Ctrl: 1.3 ± 0.1 nA, n = 10) following NO treatment (NO: 2.0 ± 0.2 nA, n = 13; p < 0.05) or synaptic conditioning (PC: 2.5 ± 0.4 nA, n = 17; p < 0.05). This NO-induced K^+^ current was suppressed by r-stromatoxin-1 (NO+Strtx: 0.3 ± 0.1 nA, n = 6), whereas 7-NI (10 μM) prevented conditioning-mediated current increases (PC+7-NI: 1.0 ± 0.2 nA, n = 6), confirming that nitrergic signaling induced a current with a more negative activation than under control conditions. Currents generated around AP threshold in CA3 neurons at −40 mV also increased following NO treatment or conditioning (Ctrl: −25 ± 80 pA, n = 9; NO: 377 ± 88 pA, n = 5; PC: 282 ± 145 pA, n = 5; p < 0.05), and this was suppressed by r-stromatoxin-1 (NO+Strtx: 82 ± 93 pA, n = 4) and by 7-NI treatment during conditioning (PC+7-NI: 107 ± 59 pA, n = 3), confirming a NO-dependent Kv2 current activation at potentials around AP threshold.

Further evidence of the conductance change was obtained by tail current measurements from the MNTB (Figures 3I and 3J) and CA3 (Figures 3K and 3L). Fit of a Boltzmann function showed that NO signaling (NO donor or PC) caused a marked leftward shift of the activation curve (V_1/2_) in neurons from both brain regions that was blocked by 7-NI or by glutamate receptor antagonism during the conditioning paradigm (Figures 3J and 3L). It is not possible to precisely equate half-activation voltages between recombinant and native K^+^ channels (because there are many unknowns in terms of heteromeric assembly, accessory proteins, and phosphorylation states), but such a leftward shift is consistent with a reduced contribution from Kv3 channels that have a more positive half-activation voltage (Hernández-Pineda et al., 1999; Kanemasa et al., 1995; Rudy and McBain, 2001) than Kv2 channels (Guan et al., 2007; Johnston et al., 2008; Kramer et al., 1998).

**Figure 4:**
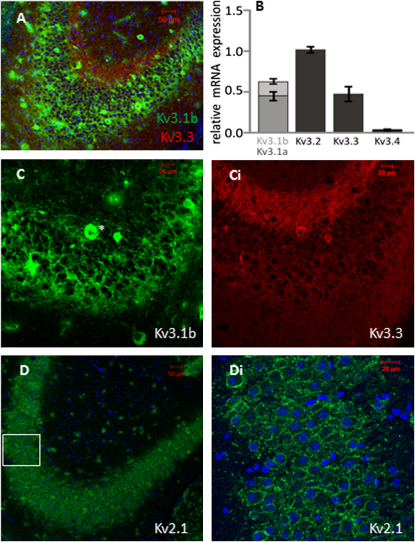
Kv3 Channel Subunits Are Expressed in CA3 Pyramidal Neurons (A) Confocal image of the CA3 pyramidal cell layer showing neurons stained with antibodies against Kv3.1b (green) and Kv3.3 (red). (B) qPCR data show relative mRNA expression of Kv3.1a/b, Kv3.2, Kv3.3, and Kv3.4 using laser microdissection of pyramidal neurons of the CA3 region. (C) CA3 pyramidal cell neurons stained with an antibody against Kv3.1b (C) and Kv3.3 (Ci); note interneuron marked with an asterisk (^∗^) and membrane staining of pyramidal cells for Kv3.1 and Kv3.3. (D) Images of the same region stained with an antibody against Kv2.1 (D, 10×; Di, 40×) also show clear plasma membrane labeling. Box area under higher magnification in Di. Cell nuclei are shown by the blue DAPI costain. Data are denoted as mean ± SEM.

Additional evidence for expression of Kv3 and Kv2.1 channels came from immunohistochemistry and qPCR experiments, showing Kv3.1b, Kv3.3, and Kv2.1 protein (Figures 4A, 4C, and 4D) and Kv3.1a, Kv3.1b, Kv3.2, and Kv3.3 mRNA (Figure 4B) in CA3 pyramidal cell bodies. We could not detect immunostaining (not shown) or substantial mRNA for Kv3.4. Together, these data confirmed that Kv3 channels are present in hippocampal CA3 pyramidal neurons as reported previously (Perney et al., 1992; Weiser et al., 1994).

We excluded significant contributions from Kv1, Kv4, and BK K^+^ channel families: Kv1 was routinely blocked with dendrotoxin-I (100 nM; data not shown); Kv4 was inactivated by the conditioning voltage of the I/V protocol (Figure S2); and the NO-potentiated current was not a BK because this was TEA insensitive. We conclude that NO signaling mediates an activity-dependent adaptation in postsynaptic excitability by suppressing Kv3 and potentiating Kv2 currents in both the brain stem and hippocampus.

**Figure 5:**
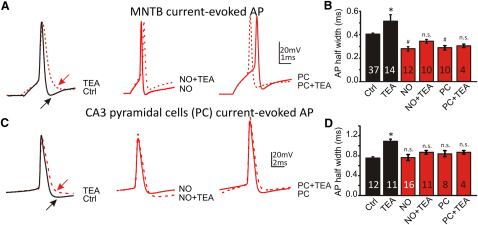
NO Signaling Suppresses Kv3 Contribution to AP Waveforms (A) MNTB. Left view shows that control APs (black trace) are fast in the MNTB and increase in duration when Kv3 is blocked with TEA (1 mM, red-dashed trace). Middle view illustrates that in the presence of NO donors, APs remain of short duration, but now TEA (1 mM, red trace) had no effect. Right view shows that after synaptic conditioning, APs are fast, and their duration is unaffected by TEA. (B) Summary of MNTB AP half-widths for the above conditions. Note that TEA significantly increases AP duration only under control conditions (black bars, ^∗^p < 0.001 versus Ctrl), and AP durations following nitrergic signaling are faster than control APs (^#^p < 0.01 versus Ctrl). (C) CA3. Left view shows that control APs (black trace, with a longer half-width than the MNTB) increase in duration when Kv3 is blocked with TEA (1 mM, red-dashed trace). Middle view illustrates that in the presence of NO donors, APs remain the same duration as control, but now TEA (1 mM, red trace) had no effect. Right view shows that after synaptic conditioning, APs are also unaffected by TEA. (D) Summary of CA3 AP half-widths for the above conditions. Note that TEA significantly increases AP duration only under control conditions (black bars, ^∗^p < 0. 0243 versus Ctrl). Statistics were determined by one-way ANOVA with posttest, unpaired data. n.s., not significant. Data are denoted as mean ± SEM.

These results suggest that neuronal delayed rectifiers are malleable; under low-activity conditions, Kv3 contributes to outward rectification, but during more active periods, Kv2 channels become dominant. This idea was tested in both MNTB and CA3 by examining the effect of TEA (1 mM) on AP waveforms under control conditions (before conditioning), on exposure to NO donors, or after synaptic conditioning (Figure 5). TEA slowed the time course of control APs (MNTB, Figures 5A and 5B; CA3, Figures 5C and 5D) but had no effect on the AP waveforms after exposure to NO or after synaptic conditioning. This clearly shows that after nitrergic activation, Kv3 channels were no longer involved in AP repolarization. Additional contributions of nitrergic signaling by suppression of voltage-gated sodium currents have been reported in the MNTB (Steinert et al., 2008). The maximal rate of rise of APs in CA3 pyramidal neurons was unaltered following activation of nitrergic signaling (NO and PC; data not shown).

### Switching MNTB Delayed Rectification from Kv3 to Kv2

Our data suggest the idea that AP repolarization could be mediated by different Kv families under different activity conditions in the same neuron. To test this hypothesis, we focused on the MNTB neuron, which has a well-documented expression of Kv3.1b and Kv2.2 subunits. The restricted expression of Kv2.2 to the brain stem (Johnston et al., 2008) also allows use of a transgenic knockout (KO) with relatively few complications, which would not be possible for a Kv2.1 KO because of its broader expression (Misonou et al., 2005). The compact size of MNTB neurons with few dendrites also assisted voltage-clamp interpretation by minimizing space-clamp issues.

**Figure 6:**
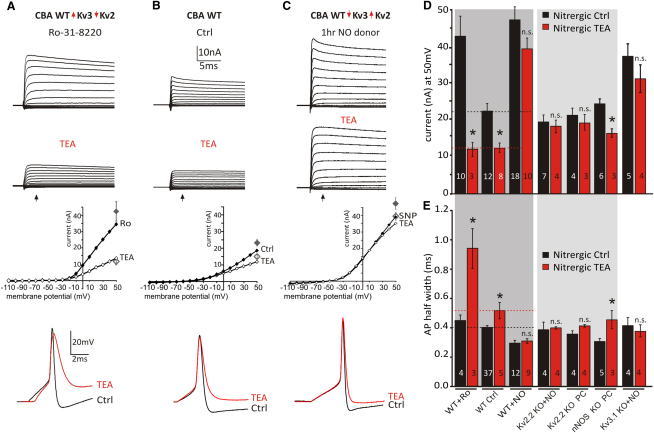
PKC and NO Signaling Switch between Kv3 and Kv2-Dominated AP Repolarization (A)–(C) show MNTB K^+^ currents under voltage clamp for three conditions: (A) with PKC blocked, (B) control, and (C) NO signaling active. For each column, raw traces are shown on top, traces in the presence of TEA (1 mM) are in the second row, the third row shows I/V relationships for the above data (measured at the latency indicated by the arrow) with average data for currents evoked at +50 mV (gray symbols). The bottom row illustrates current clamp AP waveforms under each condition, with control and TEA traces superimposed. (A) CBA mouse (WT) MNTB neuron K^+^ currents are large when PKC is inhibited by Ro31-8220 (1 μM). TEA (1 mM) shows a large block because Kv3 predominates, as shown in the I/V. Under current clamp, block of Kv3 with TEA dramatically increases AP duration. (B) The control CBA MNTB neuron (no treatment) shows basal Kv3 TEA-sensitive currents contributing around 30% of the outward K^+^ current, so TEA has a more modest effect on AP duration (bottom). (C) In neurons with nitrergic signaling activated by a NO donor, large outward K^+^ currents are evoked, but TEA has no effect on the I/V relationship nor does TEA have a significant effect on the AP time course (lower traces). (D and E) The (C) paradigm with activation of nitrergic signaling was repeated with tissue from Kv2.2 KO, Kv3.1 KO, and nNOS KO mice, and the average data are summarized in the bar graph: (D) current measured at +50 mV; (E) AP half-width. In each case mean data are plotted with (red) and without (black) TEA (1 mM). The dark-gray shading indicates WT animals, and the light-gray shading indicates data from the Kv2.2 KO and nNOS KO; n is indicated within bars. Statistics were determined by one-way ANOVA with posttest, unpaired data, ^∗^p < 0.05, n.s., not significant versus its own Nitrergic Ctrl. Data are denoted as mean ± SEM.

Under control in vitro conditions, MNTB neurons possess around 23 nA of outward K^+^ current (at +50 mV), of which TEA-sensitive Kv3 currents account for 31% (Figure 6B) (Macica et al., 2003; Wang et al., 1998). To unmask phosphorylated (inactive) Kv3 currents (Song et al., 2005), PKC antagonists were employed to block basal PKC activity (Figure 6A). Ro31-7549 (100 nM) and GF109203X (1 μM) both inhibit conventional and novel PKC-δ and PKC-ɛ isozymes (Song et al., 2005), allowing full activity of endogenous Kv3 channels to be monitored. MNTB neurons now exhibited larger outward currents of 43 ± 6 nA (at +50 mV), and TEA (1 mM) blocked 73% of outward current, consistent with increased activity of Kv3 channels. Note that in the presence of TEA, the current magnitudes in the presence of PKC antagonists were similar to CBA WT+TEA (I/V curves are shown in Figures 6A and 6B), consistent with specific action of PKC on Kv3 channels. Activation of nitrergic signaling by a NO donor also increased outward currents; but importantly, TEA now had negligible actions in suppressing this potentiated outward current (Figure 6C), and the TEA-*in*sensitive current is 3-fold larger than in control or PKC-blocked neurons. These data are consistent with a NO-dependent switch to dominance of a Kv2-delayed rectifier following sustained synaptic activity.

### The Kv3/Kv2 Activity-Dependent Switch Is Absent in MNTB Neurons from Kv2.2 KO and nNOS KO

Current clamp recordings confirmed that Kv3 made a major contribution to AP repolarization in naive MNTB neurons (Figure 6B, lower traces) because the AP half-width was increased by TEA. But *after* nitrergic activation, TEA had no effect on AP waveform (Figure 6C, lower traces), consistent with lack of Kv3. The mean current magnitude under voltage clamp (Figure 6D) and the mean AP half-width under current clamp (Figure 6E) were compared in CBA WT mice (Figure 6, dark-gray region) and three KO mice as shown in Figure S3. The contribution of Kv3 currents following nitrergic activation is indicated by the difference between the paired bar graphs: “Nitrergic ctrl” (black bars) and the “Nitrergic TEA” (1 mM, red bars), which show a significant Kv3 contribution for three conditions: control (WT Ctrl), PKC block (WT+RO), and the nNOS KO (nNOS KO PC). The TEA-sensitive current in the nNOS KO is similar to control and consistent with no nitrergic signaling (which would otherwise have suppressed the Kv3 current; Figure S3C). The pharmacological data in Figure 3 point to nitrergic potentiation of Kv2 currents and predict that NO-mediated potentiation of the K^+^ current will be absent in the MNTB from the Kv2.2 KO mice—and it is: the result in Kv2.2 KO animals is summarized in the light-gray shading of Figures 6D and 6E; where outward K^+^ currents remained small (<20 nA, no potentiation), and both current and AP waveforms were TEA insensitive, as Kv3 has been suppressed by NO (Figures S3A and S3B). Finally, we tested the K^+^ currents from the Kv3.1 KO; here, the prediction would be that nitrergic potentiation should be intact. K^+^ currents in Kv3.1 KO mice increased from 15 ± 1 nA (n = 10) to 38 ± 3 nA (n = 5) following nitrergic activity (Figure 6D, Kv3.1 KO+NO, black bar, traces in Figure S3D), confirming a non-Kv3 current potentiation that again is TEA insensitive following NO signaling (Figure 6D, Kv3.1 KO+NO, red bar). These results are all consistent with the postulated activity-dependent NO-mediated signaling pathway acting to suppress Kv3 currents and potentiate Kv2 currents.

Both Kv2 and Kv3 channels are regulated by protein phosphorylation (Macica et al., 2003; Park et al., 2006), which adapts intrinsic excitability in hippocampus (Misonou et al., 2004) and MNTB (Song et al., 2005). Basal phosphorylation of Kv3.1 is reduced by brief sound exposure or synaptic stimulation (lasting seconds), thereby slightly augmenting Kv3.1 via a PP1/PP2A-dependent mechanism (Song et al., 2005). Longer-term synaptic activity (15–25 min) suppresses Kv3 channels through NO signaling (Steinert et al., 2008), and here, we show that following sustained synaptic stimulation or NO-donor application for >1 hr, Kv3 currents remained suppressed, but Kv2 currents were facilitated. This dynamic changeover resulted in a transient increase in AP half-widths (Figure S4C). The overall time course of the nitrergic modulation of outward currents reflects the early decline in Kv3 reported previously (Steinert et al., 2008) and the slower increase in Kv2 reported here, which takes around 1 hr and is shown for Peak (Figure S4A) and Plateau (Figure S4B) currents and AP half-width (Figure S4C). Recovery was observed after 1 hr perfusion in NO-free aCSF indicating that this NO-induced potentiation of Kv2 is not related to apoptosis induction (Pal et al., 2003; Redman et al., 2007), which further suggests that MNTB neurons in brain slices kept over more than 1 hr may differ from their in vivo conditions because the slice has no ongoing spontaneous firing, and the NO-signaling cascade is diminished. In contrast, spontaneous in vivo activity leads to elevated levels of NO, which undoubtedly contribute to nitrergic signaling and, therefore, might underlie the here-observed current potentiation following synaptic conditioning.

This signaling reported here relies on phosphorylation, and inhibition of PP1 and PP2A with okadaic acid (OA; 50 nM) had no effect on Kv potentiation induced by NO donors (Figure S5A). Interestingly, inhibition of PKC (Ro31-7549 or GF109203X) completely abolished NO-induced Kv2 potentiation (Figure S5A). Thus, the NO-mediated Kv2 enhancement requires both PKC and the classical NO pathway through activation of guanylyl cyclase and PKG (Figure S5B).

### Kv2 Dominance Allows Sustained Firing at Higher Frequencies

**Figure 7:**
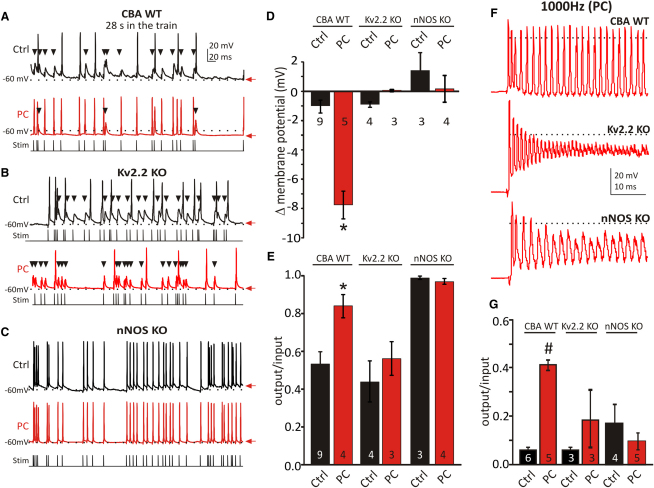
Kv2 Maintains AP Firing and Information Transmission during Sustained Activity Comparison of the postsynaptic AP firing during sustained synaptic stimulation (Stim) in WT, Kv2.2 KO, and nNOS KO mice. (A) AP firing during evoked synaptic 100 Hz train (30 s, Poisson-distributed ISIs, sample traces start at 28 s, stimulation indicated by the lines plotted below each trace). WT CBA (control, black; after conditioning, PC, red). Note the number of failures (black arrowheads) is reduced following PC. (B) Kv2.2 KO (control, black; after conditioning, PC, red) shows no improvement after synaptic conditioning. (C) nNOS KO (control, black; after conditioning, PC, red) shows high fidelity before and after synaptic conditioning. (D) Summary bar graph of membrane potential difference between beginning and end of the 30 s train, for each of the three genotypes in control (black) and following PC (red); only the WT CBA mice with intact Kv2.2 and nNOS signaling show hyperpolarization of interspike potentials (^∗^p < 0.0001). (E) Fidelity (output/input ratios) measured between 28 and 30 s shows improvement in WT CBA following PC, whereas the Kv2.2 KO shows no improvement. The nNOS KO maintains a high fidelity at 100 Hz, but transmission fails at higher frequencies as shown in (F) where 1000 Hz trains are only faithfully followed in WT CBA mice following PC, but APs fail after a few milliseconds in Kv2.2 KO and nNOS KO. (G) Summary of output/input ratios for a 1000 Hz train for the three genotypes. n is indicated within bars. Statistics were determined by one-way ANOVA with post-test, unpaired data,^∗^p < 0.02, ^#^p < 0.05. Data are denoted as mean ± SEM.

So what is the physiological relevance of switching between delayed rectifiers? Kv3 channels have fast activation and deactivation kinetics and so turn on and off quickly. Kv2 channels have slower kinetics, allowing cumulative activation during periods of high synaptic activity and leading to enhanced membrane hyperpolarization, thereby encouraging recovery of sodium channels from inactivation (Johnston et al., 2008). This suggests functional relevance as a homeostatic gain control mechanism, where Kv2 dominance improves/maintains the dynamic range of signaling with increasing activity. To test this, we examined the ability of synaptic conditioning to modulate transmission fidelity across a range of physiological frequencies during long-lasting trains of synaptic stimulation (30 s, 100 Hz Poisson-distributed ISIs). Initial firing in the 30 s train (not shown) showed high fidelity (Hennig et al., 2008), but during such long trains the firing probability declined, so that the majority of EPSPs failed to trigger APs by the end of the train (Figure 7A, black). Following synaptic conditioning, the number of failures (Figure 7A, PC, red, black arrowheads) was reduced in control CBA mice, whereas no improvement was observed in Kv2.2 KO mice (Figure 7B, PC, red). This cannot be solely explained by reduced excitability, but the observed cumulative interspike hyperpolarization of 7.9 ± 1.3 mV in WT mice (Figure 7D; p < 0.0001, one-way ANOVA with posttest) allows greater recovery of Na^+^ channels from inactivation (Johnston et al., 2008) and thereby increased output/input fidelity (Figure 7E). On the other hand, Kv2.2 KO and nNOS KO mice showed no hyperpolarization (Figures 7B–7D) and no improvement of fidelity (Figure 7E), indicating that Kv2.2 and nNOS signaling are required to allow reliable transmission across this synapse. Although low-frequency firing (100 Hz Poisson train) was well maintained in Kv2.2 KO and nNOS KO mice (Figures 7B and 7C) due to the lack of NO signaling and subsequent functional dominance of Kv3, high-frequency fidelity required Kv2.2 currents and NO signaling. High-frequency trains (1000 Hz) can be followed with the highest fidelity only in WT CBA mice PC (Figures 7F and 7G; p < 0.0001, one-way ANOVA with posttest). After an initial one-to-one response, APs followed alternate stimuli within the train. This “skipping cycle” at higher frequencies (Song et al., 2005) is likely to happen in vivo but does not occur in either KO, implicating the necessity of NO-Kv2 signaling in order to allow firing across the full physiological range of MNTB neurons (Kopp-Scheinpflug et al., 2003).

### Kv2.2 Currents Rapidly Decay following Brain Slice Preparation

**Figure 8:**
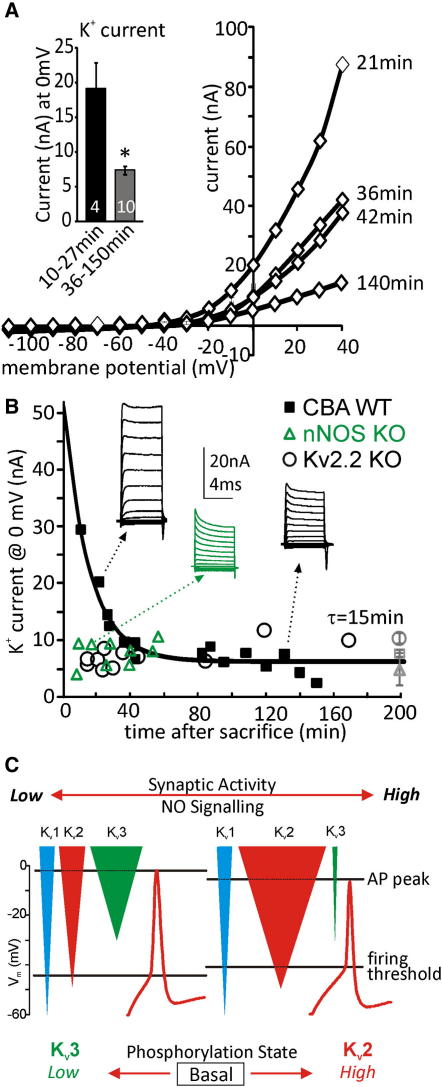
Kv2 Currents Rapidly Decay on Tissue Isolation Implying High Levels of Endogenous Signaling Rapid whole-cell patch recording from MNTB neurons showed the largest outward currents in cells recorded most recently following animal sacrifice. (A) I/V relationships from four MNTB neurons recorded within minutes of animal sacrifice; inset shows averaged K^+^ current amplitudes for indicated time periods. (B) Amplitude of Kv currents (black squares, measured at 0 mV) plotted against time from animal sacrifice. Insets show example current traces for representative time points. Triangles are data from nNOS KO and circles from Kv2.2 KO animals showing no enhanced currents at early time points. Solid line shows exponential data fit. Gray square/triangle/circle (far right) gives mean values for control and nNOS KO and Kv2.2 KO. (C) Schematic illustrating NO-dependent regulation of Kv channels. Under inactive conditions with little excitatory synaptic input, several Kv conductances contribute to the whole-cell current (shown on the left). Under control (left, quiescent) conditions, with little NO signaling, there is little contribution from Kv2. When activity increases, Kv3 and Kv2 currents become increasingly phosphorylated leading to NO-dependent suppression of Kv3 and activation of Kv2. Now there is enhanced current at firing threshold leading to reduced excitability and a 2- to 3-fold current increase resulting in faster AP repolarization. This enhanced delayed rectification is essential to maintain AP firing during high-frequency activity because the enhanced hyperpolarization favors recovery of Na^+^ channels from inactivation. Data are denoted as mean ± SEM.

Extrapolation from the above data suggests two predictions for activity-dependent enhancement of outward K^+^ currents: first, that neuronal K^+^ currents in vivo will be larger than in vitro; and second, that the in vivo outward K^+^ currents would be dominated by Kv2 rather than Kv3 currents because on isolation the brain slices lose sensory input and spontaneous firing, so endogenous nitrergic signaling is low. In vivo testing of this hypothesis was not feasible because it involves “blind” patch recording with high access resistance from an awake animal (i.e., without anesthetic). However, it is possible to sacrifice the mouse and process the tissue so rapidly that we can obtain MNTB patch recordings within a few minutes (10 min) of the animal's death (see Supplemental Experimental Procedures). Data obtained using this approach clearly showed that the fastest recordings had the largest magnitude K^+^ currents (i.e., at +40 mV: 87 nA [21 min]) across the full voltage range, and currents decreased with time in vitro, as shown by the overlaid I/V plots (Figure 8A; inset shows mean currents for short and long time periods). A plot of the current amplitude against the time from sacrifice (each data point is from a different neuron and measured at 0 mV, which is around AP peak voltage) showed that the initial large currents decayed with a time constant (τ) of 15 min to values previously considered “normal” under “control” in vitro conditions in resting slices (∼23 nA; I/V current traces are shown in Figure 8B inset). Repeating the experiment using tissue from nNOS KO (green triangles) or Kv2.2 KO (white circles) showed no potentiated currents at early time points, confirming that the larger WT currents required nNOS signaling and Kv2.2 channel subunits. The mean data from neurons after more than 1 hr post-sacrifice are shown on the far right (Figure 8B, gray square, WT CBA; gray triangle, nNOS KO; gray circle, Kv2.2 KO) and reflect previous control currents.

Together, our data showed that enhanced Kv2 currents reduce neuronal excitability (Figures 1–3), induce shorter AP waveforms due to stronger repolarization, and subsequently lead to interpotential hyperpolarization (in the MNTB, Figure 7), which results in enhanced AP fidelity across a wider range of physiological synaptic transmission frequencies. These data further suggest that extrapolation of our current understanding of “resting conductance” needs to be reconsidered in the light of activity-dependent modulation of intrinsic ionic conductances.

## Discussion

These results demonstrate that excitatory synaptic inputs control target neuron intrinsic excitability through reciprocal modulation of different voltage-gated K^+^ channels via nitrergic-signaling pathways in both the brain stem and hippocampus. Under low synaptic activity conditions, Kv3 currents contribute to AP repolarization, but following sustained moderate synaptic activity (within normal ranges for an awake animal in vivo), NO signaling suppresses Kv3 and enhances Kv2 currents, so that the basis of delayed rectification is then dominated by Kv2 (Figure 8C). This nitrergic modulation declines with a time constant of 15 min on isolation of brain tissue, suggesting that our estimates of “normal” K^+^ currents based on data from quiescent in vitro brain slices need to be revised. We conclude that this mechanism of postsynaptic plasticity adapts target neuron excitability and information transmission to the ongoing synaptic activity. This phenomenon complements other forms of synapse-specific plasticity and synaptic scaling, adding a new dimension to the interplay between synaptic strength and target response.

### Methodological Considerations: Why Have These Observations Not Been Made Before?

Recording with low access resistance and correction for series resistances is crucial when recording large currents (>5 nA), but it is inevitable that currents evoked along a cable structure (Williams and Mitchell, 2008) are underestimated when measured at the soma. Additionally, whole-cell recording and dialysis of the cytoplasm rapidly extinguish NO signaling (Wilson and Garthwaite, 2010), but the high series resistance of perforated patch recording to avoid dialysis makes it impossible to voltage clamp large conductances. So, most previous whole-cell patch recording (including our own) would not have detected the changes observed here. Therefore, the use of “unpaired” recording is an advantage over “paired” experiments (these terms are used in the statistical sense: control and test data are from different neurons). This recording mode maintains intracellular signaling by avoiding dialysis until the moment of membrane rupture. These simple and logical adaptations to patch-clamp methods clearly show that activity-dependent changes in neuronal excitability are occurring over time periods of around 1 hr.

### Delayed Rectifiers in MNTB and CA3 Pyramidal Neurons

These results bring us closer to understanding broader principles guiding function of voltage-gated K^+^ channels in neurons. The identification of *native* Kv currents (in real neurons) with respect to their recombinant counterparts is a major constraint in understanding the roles of voltage-gated K^+^ channels. We have focused on the largest currents (mediated by Kv2 and Kv3) because they dominate membrane repolarization. MNTB and CA3 pyramidal neurons are well characterized, so whereas both express other K^+^ channels (e.g., Kv1, Kv4, and Kv7), the small conductance or slow kinetics of these Kv renders their contribution secondary to the central task of AP repolarization. Obviously, the morphology, synaptic inputs, and function of MNTB and CA3 pyramidal neurons differ, but AP repolarization and its maintenance are fundamental to neuronal excitability and information transmission.

The association of Kv3 channels with fast spiking interneurons (Lien and Jonas, 2003; Rudy and McBain, 2001) does not preclude expression in CA3 pyramidal neurons, as is clear from in situ hybridization studies (Allen Brain Atlas; Supplemental Experimental Procedures) and PCR experiments showing that Kv3.1/3.2/3.3 mRNA is present in CA3 pyramidal neurons (Perney et al., 1992; Weiser et al., 1994), as confirmed by our PCR and immunohistochemistry data (Figure 4). Kv2.1 is a prominent delayed rectifier of cortex and hippocampus (Du et al., 2000; Guan et al., 2007; Murakoshi and Trimmer, 1999); Kv2.2 shows lower expression levels in cortical regions but is highly expressed in certain auditory nuclei (Johnston et al., 2008). Interestingly, both Kv2.1 and Kv2.2 show localization to the initial segment in *native* neurons (Johnston et al., 2008; Sarmiere et al., 2008), suggesting a common role in regulating excitability; although clustering at cholinergic synapses and cell bodies is also important for other roles (Misonou et al., 2004; Muennich and Fyffe, 2004).

### Role of Kv2 Current Potentiation

CA3 pyramidal neurons in vivo show a majority of single spiking responses in awake animals (Tropp Sneider et al., 2006), with only 20% of events giving a burst firing response. Spontaneous firing rates are in the range of 0.2 Hz in urethane anesthetized mice (Hahn et al., 2007), but spike trains from freely moving rodents can range between 4 and 62 Hz (Fenton and Muller, 1998; Klyachko and Stevens, 2006). As we demonstrate, potentiation of Kv2 favors single spiking (see Figure 2) in the hippocampus and would contribute to activity-dependent suppression of after-depolarizing potentials observed in vitro (Brown and Randall, 2009). Indeed, the mediation of Kv2 potentiation by NMDAR/nitrergic signaling seen here suggests that the commissural associative pathways (DCG-IV insensitive EPSCs activated under our conditions, Figure S1C), which express high levels of NMDAR (Fukushima et al., 2009; Rajji et al., 2006), may have a direct role in switching between CA3 pyramidal neuron single spiking and burst firing. This is consistent with increased CA3 pyramidal neuron excitability following genetic ablation of NMDAR (Fukushima et al., 2009) in the CA3 region.

The dominant subunit of the MNTB Kv3 channel is Kv3.1b (Macica et al., 2003), which is basally phosphorylated (Song et al., 2005) and following moderate periods of activity, becomes dephosphorylated and active. Our observations extend the concept of activity-dependent regulation of K^+^ currents over longer time periods, to when Kv3 is inactivated and Kv2 channels dominate MNTB excitability. It seems unlikely that this Kv2 enhancement is due to insertion or trafficking of Kv2 protein because immunohistochemistry shows no difference in the Kv2 distribution in *naive* and NO-treated slices (data not shown). The increase in Kv2 current amplitudes maintains or accelerates AP repolarization (in the MNTB) and is TEA insensitive in both brain regions. The MNTB exhibits some of the highest firing frequencies (>1 kHz) in the brain (Kopp-Scheinpflug et al., 2003), and transmission failure is a major problem for auditory processing (Grothe et al., 2010; Kopp-Scheinpflug et al., 2011). At these high frequencies the summed EPSPs generate sufficient depolarization and, hence, accumulation of Na^+^ channel inactivation to cause transmission failure that is opposed by the increase in Kv2-delayed rectifier currents reported here. Kv2 channels have lower activation thresholds of around −40 mV, half-activation voltage of −9 to −2 mV, and slower kinetics (Guan et al., 2007; Johnston et al., 2008; Kramer et al., 1998) that allow cumulative activation during periods of high-frequency firing and provide additional membrane hyperpolarization, promoting enhanced recovery of Na^+^ channels from inactivation (Johnston et al., 2008). Therefore, an increase in Kv2 current may lead to a more efficient repolarizing current at voltages around AP peaks. In addition the multiple Kv2 phosphorylation sites allow this channel to be modified and fine-tuned in a more complex way (Misonou et al., 2004; Mohapatra et al., 2008; Rivera-Arconada and Lopez-Garcia, 2010) than that reported for Kv3.

### An Activity-Dependent, Glutamate, and NO Homeostatic-Signaling Pathway

Glutamatergic signaling is tightly coupled to nNOS activation in both the hippocampus (Garthwaite, 2008) and brain stem (Steinert et al., 2008). In the brain stem, NMDARs are of small magnitude on maturation (Joshi and Wang, 2002; Steinert et al., 2010b) and are coupled to nNOS, but additional nNOS activation is mediated through calcium-permeable AMPARs that are dominated by GluRD subunits (Geiger et al., 1995; Youssoufian et al., 2005). Coupling between NMDAR and nNOS is generally ensured through their mutual PDZ binding in the postsynaptic density (Brenman et al., 1996), but this may be of secondary importance in the MNTB because the nNOSβ-spliced variant, which lacks the PDZ-binding motif, is also expressed in the brain stem (Eliasson et al., 1997). nNOS is widely expressed in the cortex and hippocampus, including Ivy cells (Fuentealba et al., 2008; Tansey et al., 2002; Tricoire et al., 2010), but the mobility of NO and action as a volume transmitter (Artinian et al., 2010; Garthwaite and Boulton, 1995; Steinert et al., 2008) allows regulation of neighboring neurons (up to 60–100 μm distance), which may not themselves generate NO.

### Suppression of Kv3 and Potentiation of Kv2 Currents Are Mediated by Phosphorylation

Both Kv2 and Kv3 channels are regulated by protein phosphorylation (Misonou et al., 2004; Mohapatra et al., 2008; Song et al., 2005). Basal PKC phosphorylation of Kv3.1 is reduced by brief sound exposure or synaptic stimulation (lasting seconds), thereby augmenting Kv3.1 via a PP1/PP2A-dependent mechanism (Song et al., 2005). Longer-term synaptic activity (over 15–25 min) suppresses Kv3 channels through NO signaling (Steinert et al., 2008), and here, we have shown that sustained synaptic stimulation for 60 min or more facilitates Kv2 currents, whereas Kv3 currents remain suppressed. The facilitation of Kv2 currents required activity of both PKC and PKG, but not phosphatases. Multiple sites of Kv2.1 C-terminal phosphorylation cause a proportional shift in the voltage dependence of activation of Kv2.1 (Park et al., 2006), and here, we show that cGMP/PKG signaling also modulates Kv2, perhaps indicative of alternate nitrergic phosphorylation sites, which will be explored in future studies.

This new mechanism for physiological regulation of K^+^ channel activity is important for our understanding of brain physiology. It shows that spontaneous and moderate synaptic excitation influences target neuron excitability and will complement synapse-specific forms of modulation. The result is important for integrating knowledge of single neurons and network behavior in vitro and after isolation from sensory input because our results show that ion channel activity can undergo substantial changes over periods of minutes to hours.

## Experimental Procedures

### Electrophysiology

Brain slices were prepared from P12 to P16 CBA/Ca mice, which were killed by decapitation in accordance with the Animals, Scientific Procedures Act, 1986. Transverse slices containing MNTB (200 μm) were cut in low-sodium artificial CSF (Supplemental Experimental Procedures) at ∼0°C. To identify neurons with intact calyceal synaptic connections, a calcium-imaging technique was used. Horizontal hippocampal slices of 250 μm thickness were prepared as described previously (Brown and Randall, 2005) (Supplemental Experimental Procedures). Hippocampal EPSCs were insensitive to DCG-IV (Figure S1C) (Lawrence et al., 2004), suggesting predominantly commissural inputs. Whole-cell recordings were made from identified neurons, visualized with 60× objectives on a Nikon FS600 microscope fitted with differential interference contrast (DIC) optics using a MultiClamp 700B amplifier and pClamp 9.2 software (Molecular Devices), sampling at 50 kHz, and filtering at 10 kHz. Patch pipettes were pulled from filamented borosilicate glass (GC150F-7.5; Harvard Apparatus, Edenbridge, UK) with a 2-stage vertical puller (PC-10; Narishige, Tokyo, Japan). Pipettes (2.5–3.5 MΩ) were filled with a solution containing: KCl 110 mM, HEPES 40 mM, EGTA 0.2 mM, MgCl_2_ 1 mM, CaCl_2_ 0.1 mM, Na_2_phosphocreatine 5 mM, and L-arginine 1 mM; pH was adjusted to 7.2 with KOH. Current-voltage (I/V) relationships were measured before and after synaptic conditioning (PC) in separate populations of neurons to avoid neuronal dialysis during the 1 hr conditioning period. Final whole-cell access resistance was <7 MΩ, and series resistance was routinely compensated by 70% (10 μs lag). Displayed raw traces are not corrected for leak or capacitive currents but are low-pass Bessel filtered (2 kHz). Slices for rapid recordings from MNTB neurons were prepared in an identical manner to above, except that the slice was placed in the recording chamber as soon as it was cut. All recordings were taken at physiological temperature (36°C ± 1°C).

### Immunohistochemistry

Brains were frozen in “Lamb OCT” compound (Thermo Fisher Scientific) and cryostat sectioned at 12 μm in the transverse plane. Sections were incubated with primary Abs to Kv3.1b (1:1000; NeuroMab), Kv3.3 (1:1000; Alomone), Kv3.4 (1:100; Alomone), Kv2.1 (1:100; Alomone), and diluted in PBS-T containing 1% BSA and 10% NGS overnight at 4°C. After three washes in PBS-T, sections were incubated with secondary Abs (1:1000, Invitrogen; Molecular Probes anti-goat Alexa Fluor 488 and 546 depending on primary Ab), and diluted in PBS-T, 1% BSA, and 10% NGS for 2 hr at room temperature. Images were acquired with a Zeiss laser-scanning confocal microscope (LSM 510; Carl Zeiss International).

### Quantitative PCR

Tissue samples from the CA3 soma region of the hippocampus were excised from the same batch of frozen cryostat sections used for immunostaining using laser microdissection (PALM laser system; Zeiss). PCR primers were designed using the Primer Express Software version 2.0 program (Applied Biosystems, Foster City, CA, USA). Primers were designed to cross exon-exon regions, and the gene of interest was normalized against a housekeeping gene (β-actin) (see Supplemental Experimental Procedures).

### Statistical and Data Analyses

Statistical analyses utilized unpaired two-tailed Student's t test and analysis of variance (ANOVA) with posttest to test for significance at p < 0.05. Data were tested for normality distributions. Data are denoted as mean ± SEM; “n” indicates number of neurons tested. Activation plots were fit by a Boltzmann function (I = I_max_/(1+exp(V-V_1/2_/k)), with variables I_max_, V_1/2_, and k (the slope factor). Fits were performed using Clampfit 9.2 (Molecular Devices) or Excel (Microsoft) with least-squares minimization. Input resistance (R_s_) was determined using Ohm's law applied to the voltage deflection in response to a 180 ms injection of 50 pA hyperpolarizing current applied at the resting potential. The membrane time constant was determined from a single exponential fit to the membrane-charging curve.

## Supplemental Information

Document S1. Five Figures and Supplemental Experimental Procedures
