# Supplementary material for: Nitric Oxide Is an Activity-Dependent Regulator of Target Neuron Intrinsic Excitability
Source: Neuron. 2011 Jul 28;71(2-3):291–305. doi: 10.1016/j.neuron.2011.05.037 (PMC3245892; doi:10.1016/j.neuron.2011.05.037)
Supplement: Document S1. Five Figures and Supplemental Experimental Procedures [file mmc1.pdf]

Neuron, volume *71*

Supplemental Information

Nitric Oxide Is an Activity-Dependent Regulator of Target Neuron Intrinsic Excitability

Joern R. Steinert, Susan W. Robinson, Huaxia Tong, Martin D. Haustein, Cornelia Kopp-Scheinpflug, and Ian D. Forsythe

Supplementary Information

Sections:

1. Supplemental Figures 1-5
2. *In situ* hybridisation images from the Allen Brain Atlas (Allen Mouse Brain Atlas. Seattle (WA): Allen Institute for Brain Science. ©2009; http://www.brain-map.org) (Lein et al., 2007)
3. Supplemental Methods
4. References

Supplemental Figures:

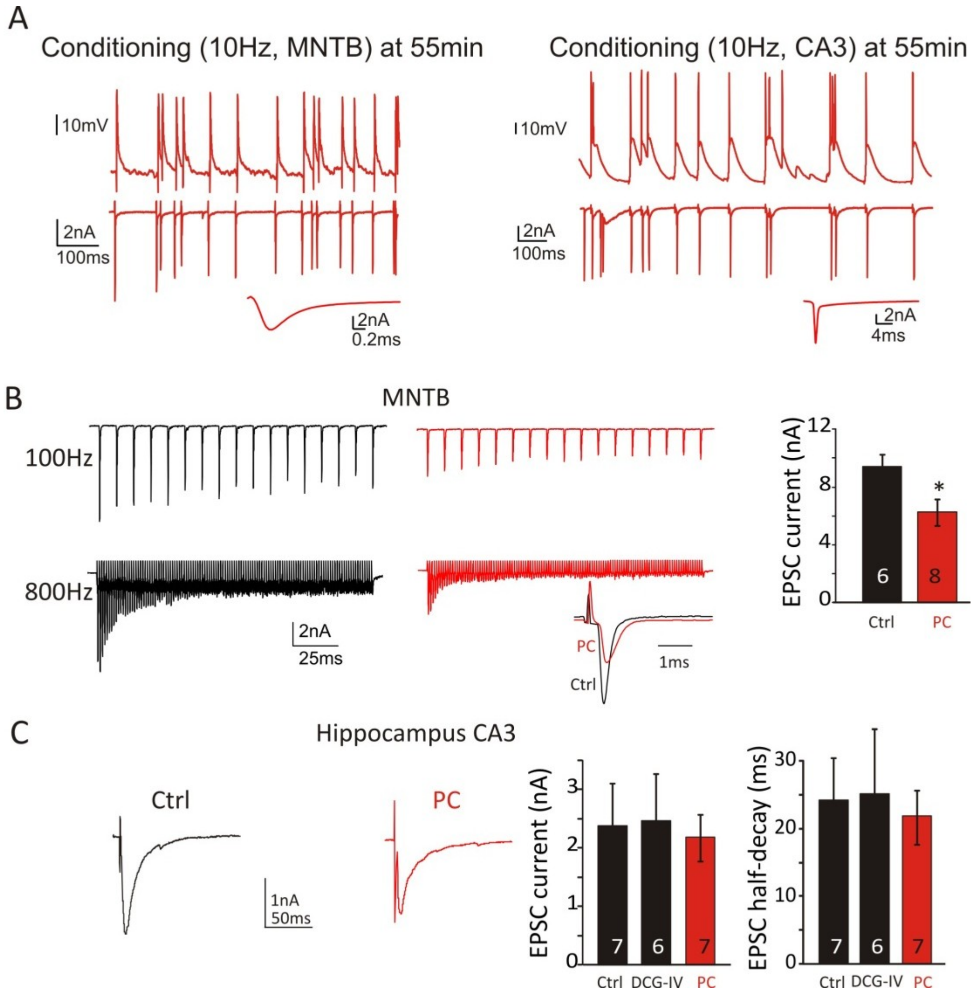

Figure S1. Conditioning (PC) does not affect hippocampal EPSCs but reduces MNTB EPSC amplitudes (relates to Fig. 1&2).

A, Conditioning trains (10Hz, Poisson ISI, for 1h) were delivered for 1h, and shown here at 55min of continuous stimulation (left for MNTB, right for hippocampus, upper current clamp; lower voltage clamp). Insets show averaged EPSCs, note the hippocampal EPSC is also showing unclamped Na^+ currents.

B, MNTB: Voltage clamp recordings of synaptic EPSCs at 100 or 800 Hz stimulus frequencies for control (black) and post conditioning (PC, red) in different neurons. Summary graph shows that mean EPSC amplitudes are reduced (inset shows slower decays kinetics following PC). *P=0.0076, unpaired data.

C, Hippocampus*:* Traces show voltage clamp recordings from CA3 neurons under Ctrl (black) and post conditioning (red) of synaptic stimulation. DCG-IV had no effect on EPSC amplitudes. Right, summary graph of EPSC amplitudes and half-decay, unpaired data.

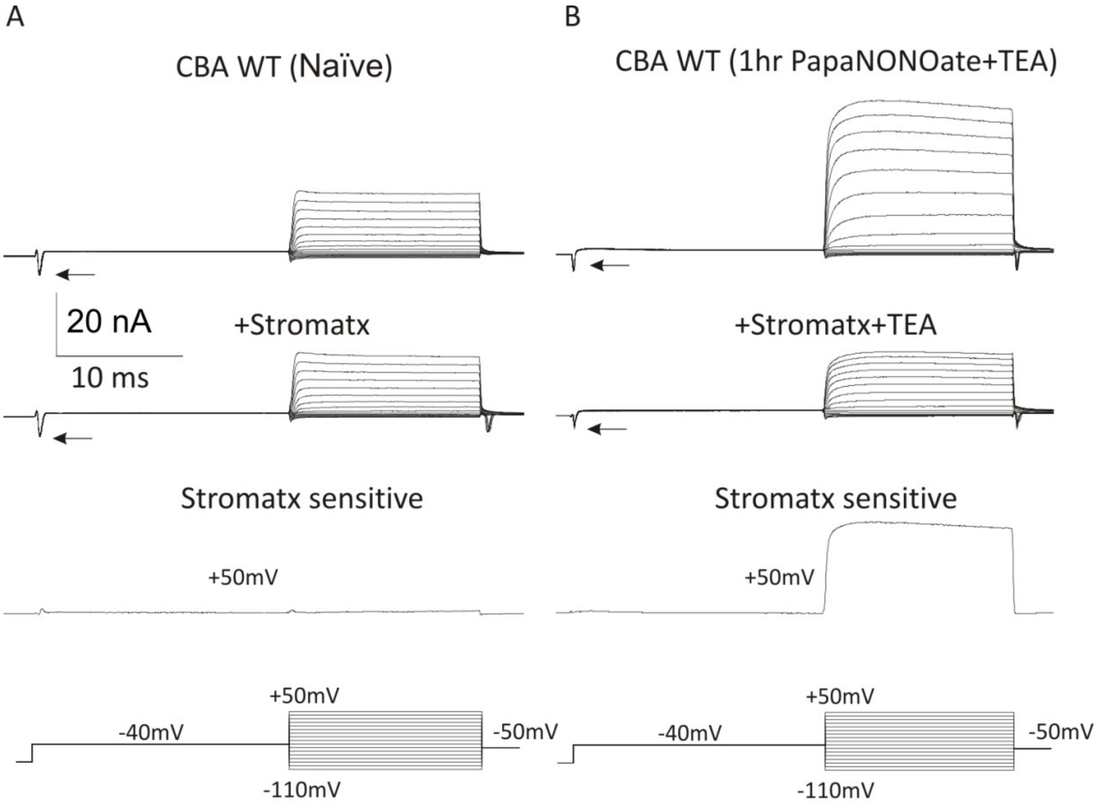

Figure S2. NO-mediated potentiated Kv currents are sensitive to r-stromatoxin-1 (relates to Fig. 3). The voltage-clamp commands are shown below (bottom row) with current traces for Naïve and NO-donor exposed MNTB neurones shown above: Top is control, Row 2 is in the presence of r-stromatoxin-1 (300nM) and row 3 shows the difference trace for +50 mV command. Note Na^+ (arrow) currents remain unchanged following antagonist application.

A, Control conditions (naïve slices) Kv currents in MNTB neurons show no sensitivity to r-stromatoxin-1 (subtracted current at +50mV) (Johnston et al., 2008).
B, NO donor application for 60min (in the presence of 1mM TEA) induced a r-stromatoxin-1 - sensitive current in MNTB neurons (subtracted current at +50mV).

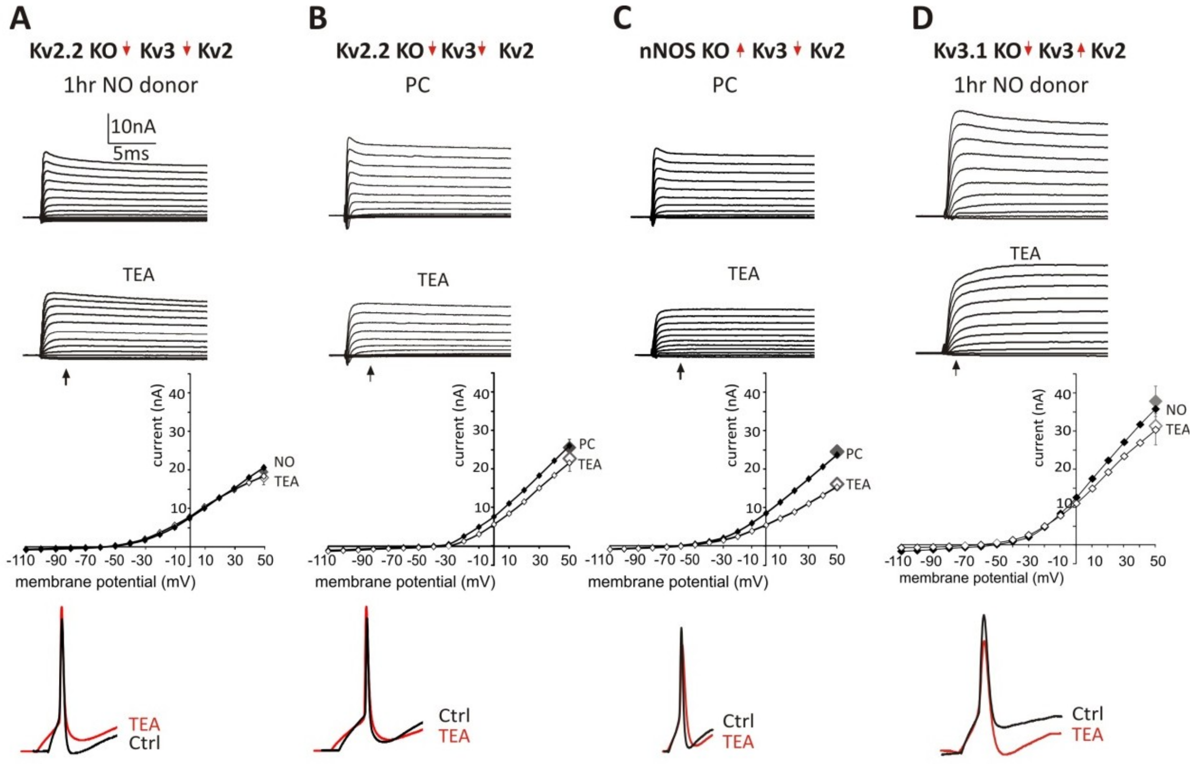

Figure S3. NO-mediated Kv potentiation requires expression of Kv2.2 and nNOS in the MNTB (relates to Fig. 6).

Each column shows I/V examples from a single neuron (with raw traces plotted above) for control and during perfusion of 1mM TEA (open symbols). Grey data points at +50mV represent means±SEM of 3-9 cells. Below, current-evoked APs are shown for indicated conditions.

A, Kv2.2 KO, 1hr after NO-donor (NO donor: 100μM SNP or 100μM PapaNONOate) perfusion. Following 1hr of NO-donor incubation, currents are reduced and TEA-insensitive, similar to NO-mediated suppression of Kv3 currents following short-term NO signalling (Steinert et al., 2008). TEA has no further effect on AP halfwidth.

B, Kv2.2 KO, Following PC, currents are reduced and TEA-insensitive, consistent with NO-mediated suppression of Kv3 currents and lack of Kv2.2 upregulation. TEA has no further effect on AP halfwidth.

C, nNOS KO, Kv currents in MNTB from nNOS KO are not potentiated following 1hr of synaptic conditioning (PC) but show a TEA sensitivity indicating that Kv3 currents are present and not suppressed as would be expected in the absence of NO signalling. APs show sensitivity to TEA indicating Kv3 contribution to repolarisation.

D, Kv3.1 KO, 1hr after NO-donor (NO donor: 100μM SNP or 100μM PapaNONOate) perfusion. Currents are potentiated but remain TEA-insensitive showing that NO-induced currents are not carried by Kv3.1. TEA has no further effect on AP halfwidth.

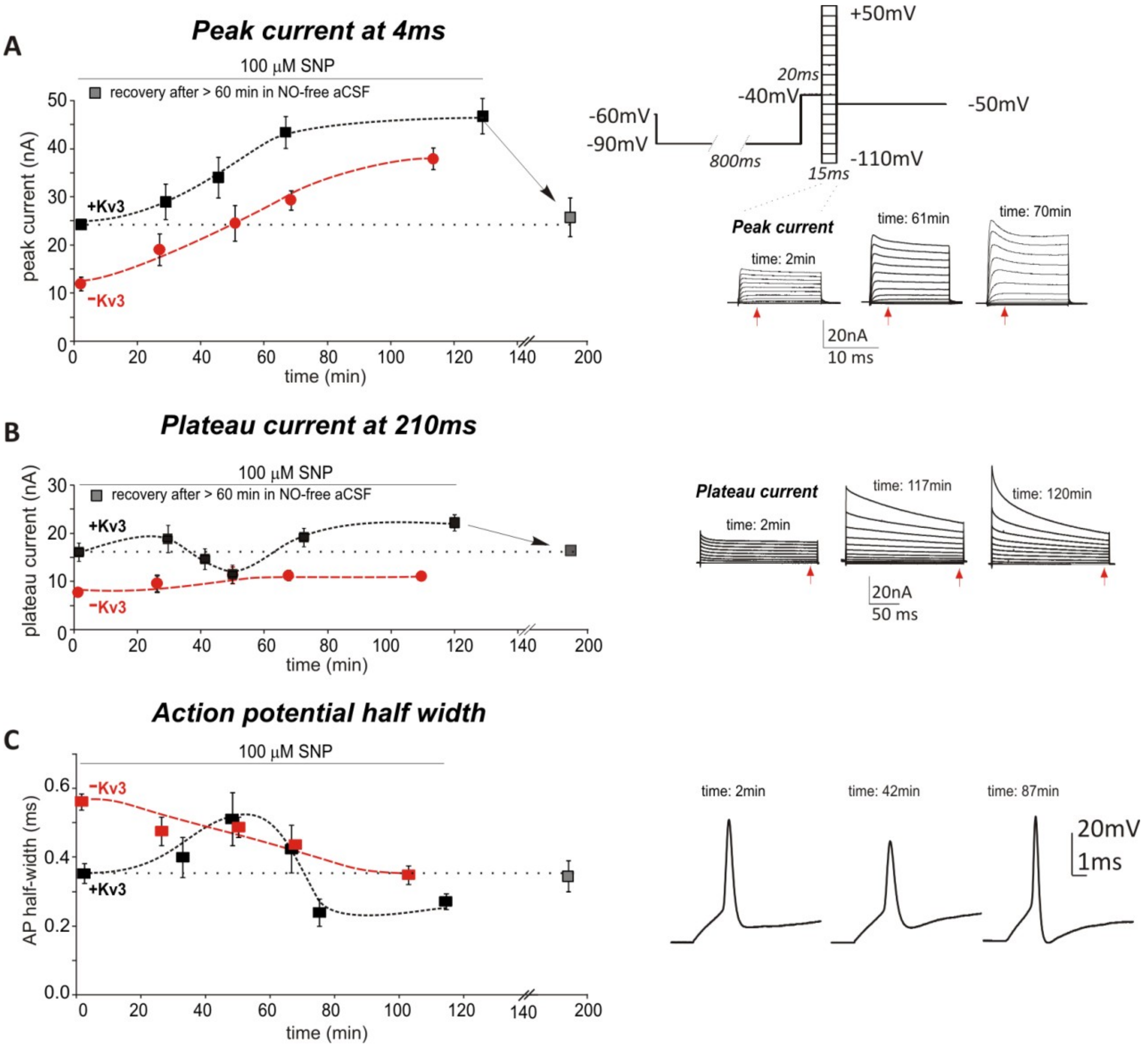

Figure S4. NO reversibly potentiates non-Kv3 currents and transiently increases AP waveforms over 60 minutes. The time course of Kv modulation here was assessed over ~2h following NO-donor incubation (SNP, 100μM; without Bay 41-2272). Short (15ms) voltage steps were used to measure PEAK current amplitudes. Long voltage steps (200ms) were used to measure PLATEAU currents (voltage command show in top inset). The peak currents increased in amplitude over the initial 60min and then on ceasing NO perfusion decayed back to control levels over 1hr. In the presence of TEA (1mM, red, -Kv3) currents were still potentiated by SNP.

A, Peak potassium currents (*4ms* latency in the step, indicated by red arrows, right traces, voltage protocol above) are plotted over time of incubation with SNP (100μM). Peak currents increase (black, +Kv3) but after incubation in SNP-free aCSF for more than 60min current amplitudes reversed to control conditions (grey square). Raw peak current traces are shown at

the indicated times to the right. In the presence of TEA (red, -Kv3) currents increase following SNP exposure suggesting that the enhanced current is not Kv3.

B, Plateau potassium currents (*210ms* latency in the step, measured at red arrows, right traces) are plotted over time of incubation with SNP. This measure of the current amplitude is very sensitive to the degree of inactivation exhibited by the dominant current (which changes as Kv3 decreases and Kv2 increases) so there is an initial decline and then increase in the current amplitude over time (black, +Kv3). After washing in SNP-free aCSF for more than 60min, current amplitudes reversed to control. Raw plateau current traces are shown at the indicated times to the right. Data denote means±SEM of 5-7 cells per data point. Red traces (plateau currents in the presence of TEA) show little change over time indicating that inactivated currents are not affected by NO signalling.

C, AP halfwidth increases over time in NO (0-50min) but strongly decreases within the next 20min of NO presence. AP halfwidth in the presence of TEA (red trace) decreased with time, rendering AP repolarisation independent of Kv3. Raw AP traces are shown at the indicated times to the right.

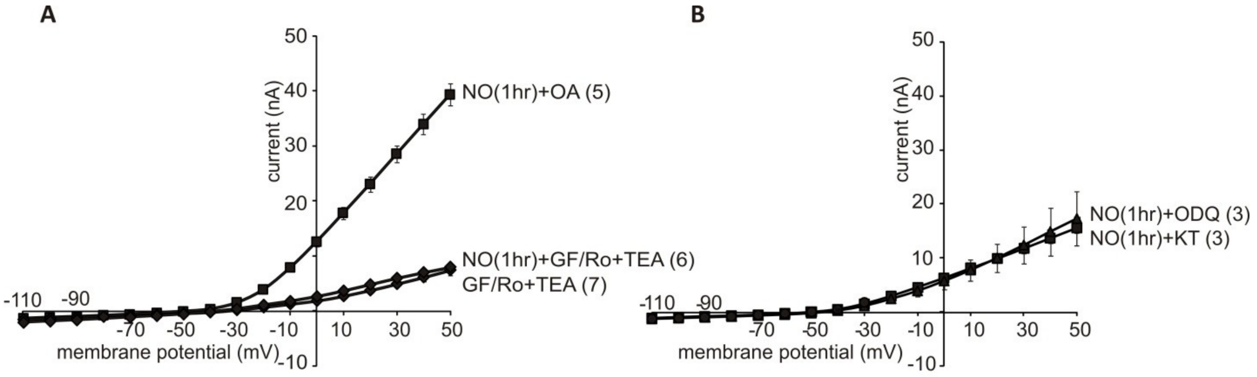

Figure S5. NO-mediated Kv potentiation requires PKC, cGMP and PKG signalling.

A, Phosphatase inhibition by okadaic acid (OA, 50nM) does not prevent NO-mediated (NO donor: 100μM SNP or 100μM PapaNONOate) Kv potentiation, whereas inhibition of PKC-δ and PKC-ε isozymes and conventional PKCs by 100nM Ro31-7549 or 1μM GF109203X blocks Kv2.2 potentiation. Inhibition of PKC also reduces basal TEA-insensitive currents ('GF/Ro+TEA' compare to Fig. 3C 'Ctrl+TEA'). Effects of PKC inhibition were measured in the presence of 1mM TEA to block any masking effects of PKC modulation on Kv3.1 (Song et al., 2005).

B, NO-mediated (NO donor: 100μM SNP or 100μM PapaNONOate) potentiation of Kv requires sGC and PKG signalling since inhibition of either enzyme by 1*H*-[1,2,4]oxadiazolo[4,3,-*a*]quinoxalin-1-one (ODQ, 1μM) or KT5823 (KT, 1μM), respectively blocked current potentiation. Data represent means±SEM of 3-6 cells each.

In situ hybridisation of nNOS, Kv2.1, Kv2.2 and Kv3 in mouse Hippocampus and MNTB (according to The Allen Brain Atlas, Allen Institute for Brain Science) (Lein et al., 2007)

nNOS Hippocampus

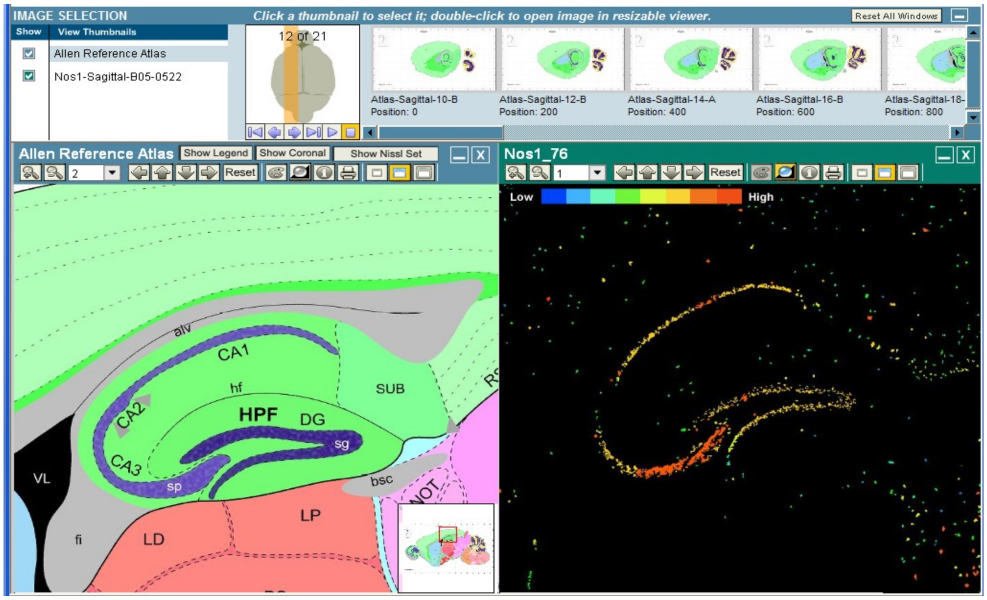

Sagital section of the Hippocampus shows strongest nNOS expression in CA3 neurons.

nNOS MNTB

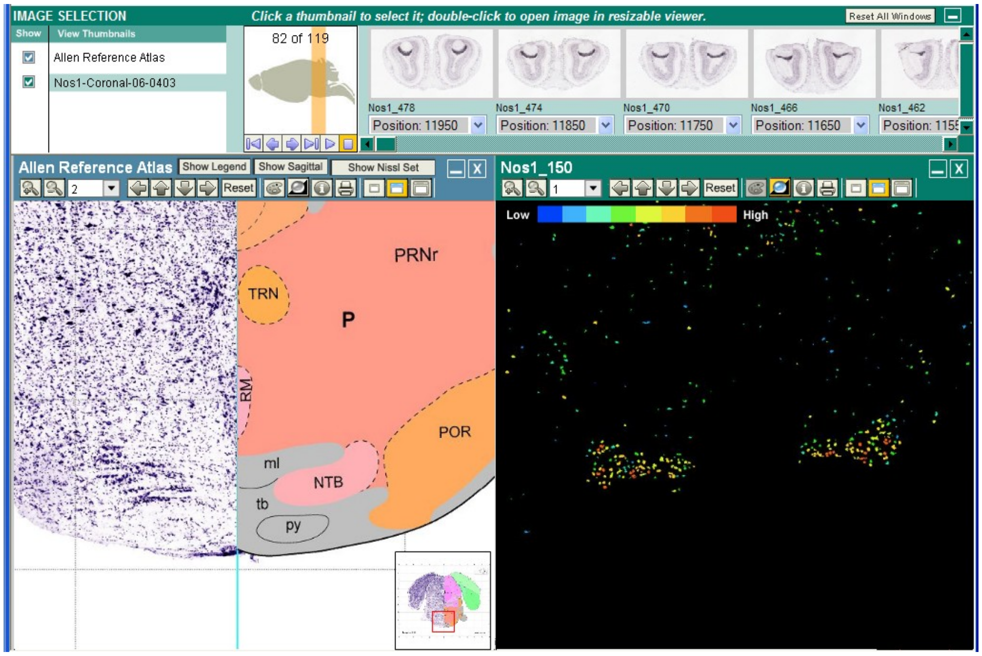

Coronal section of the MNTB shows strong nNOS expression in principal neurons.

Kv2.1 Hippocampus (very week staining for Kv2.2, see inset top right corner)

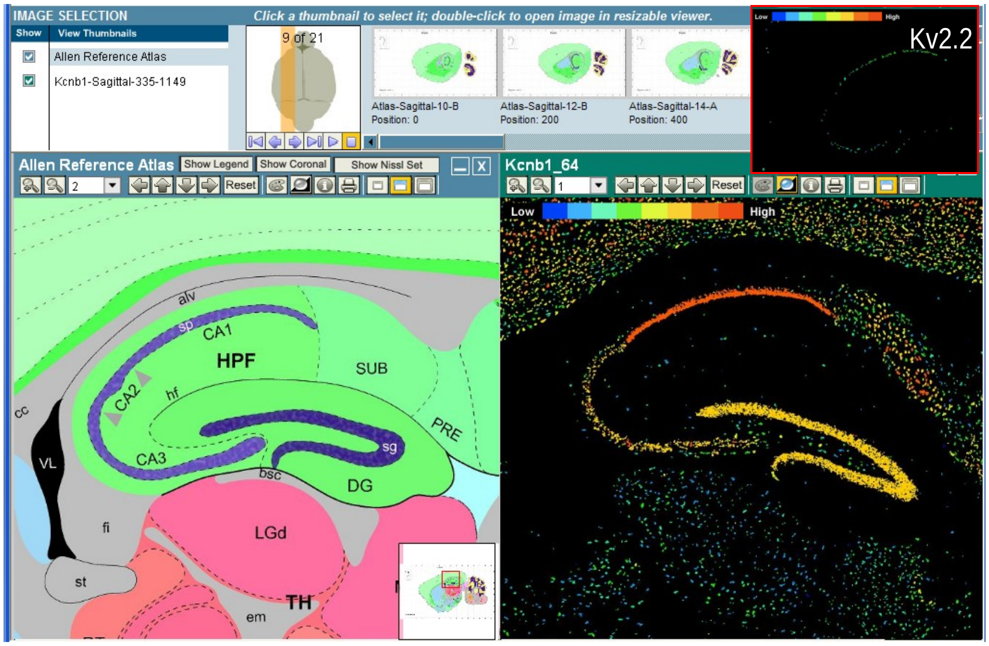

Sagital section of the Hippocampus shows strongest Kv2.1 expression in CA1 neurons but also in CA3 neurons.

Kv2.2 MNTB (no evidence for Kv2.1)

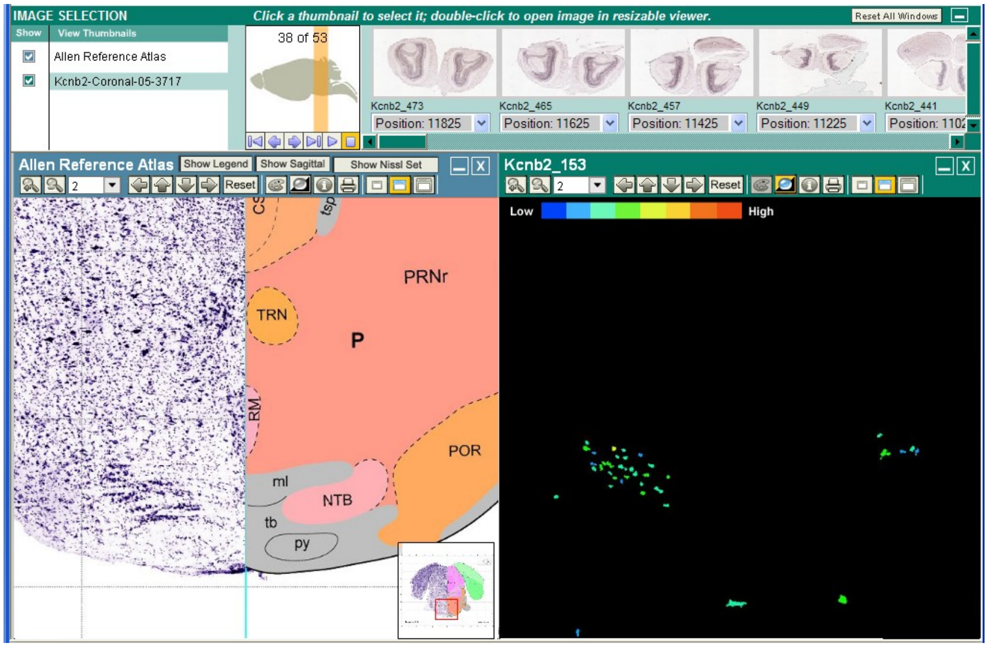

Coronal section of the MNTB shows some Kv2.2 expression in principal neurons.

Kv3.1 Hippocampus

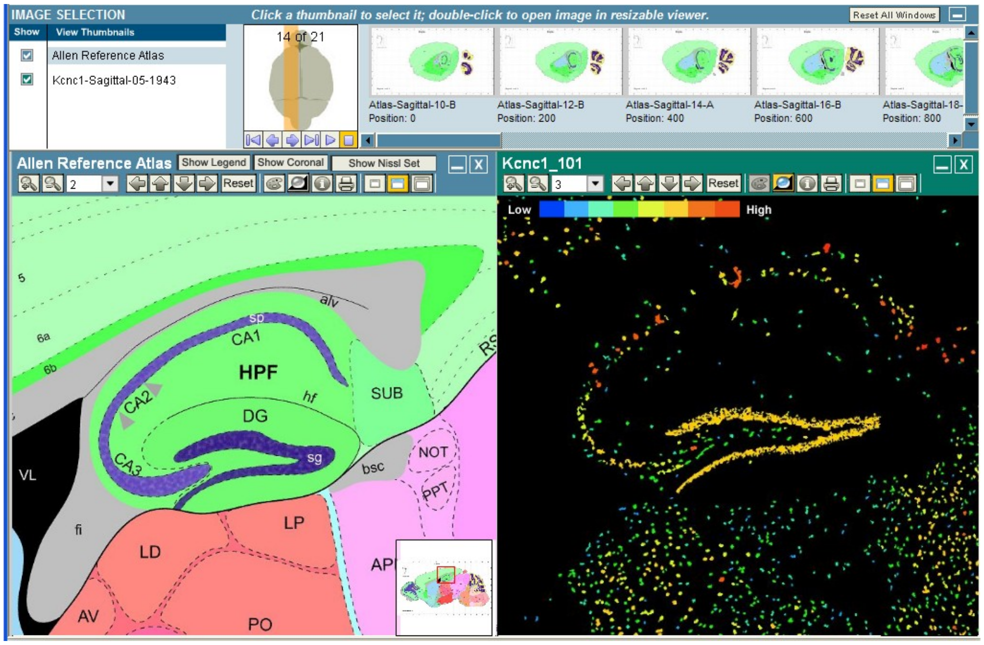

Sagital section of the Hippocampus shows strong Kv3.1 expression in CA3 neurons.

Kv3.1 MNTB

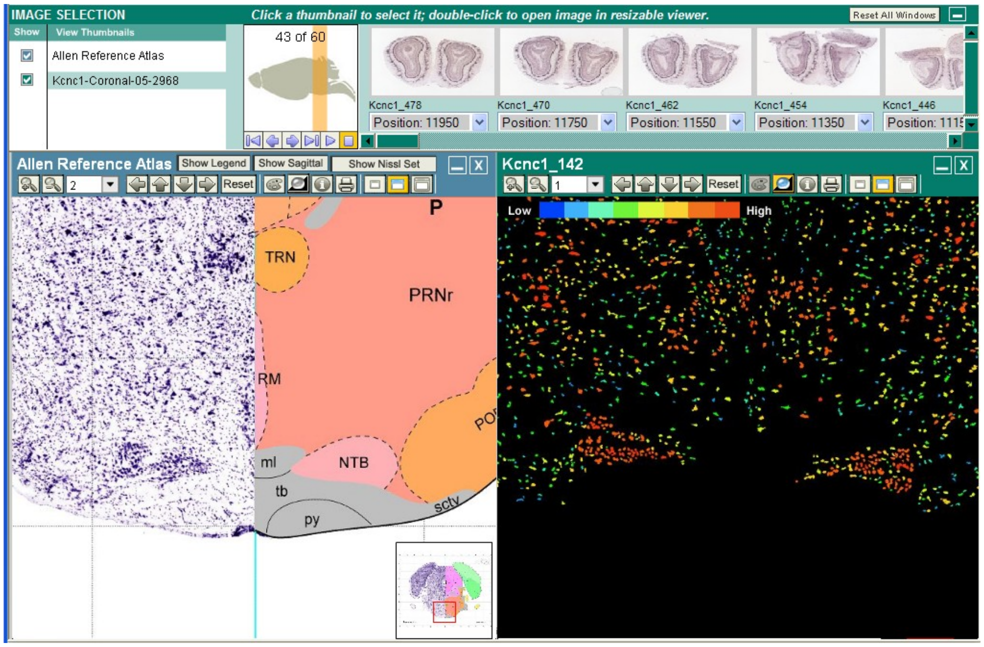

Coronal section of the MNTB shows very strong Kv3.1 expression in principal neurons.

Kv3.3 Hippocampus

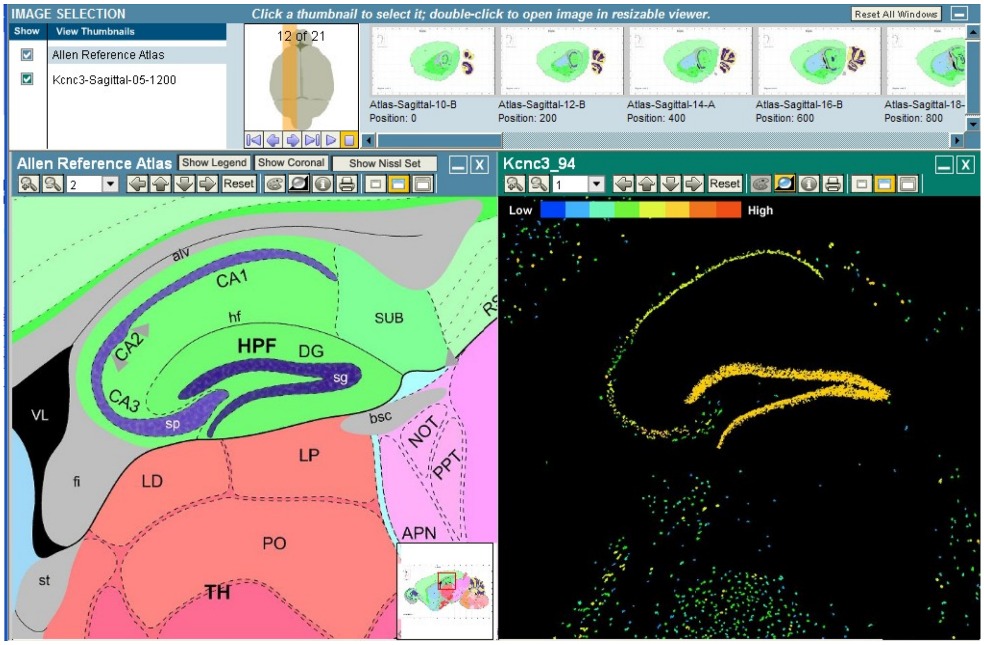

Sagital section of the Hippocampus shows strong Kv3.3 expression in CA3 neurons.

Kv3.2 Hippocampus

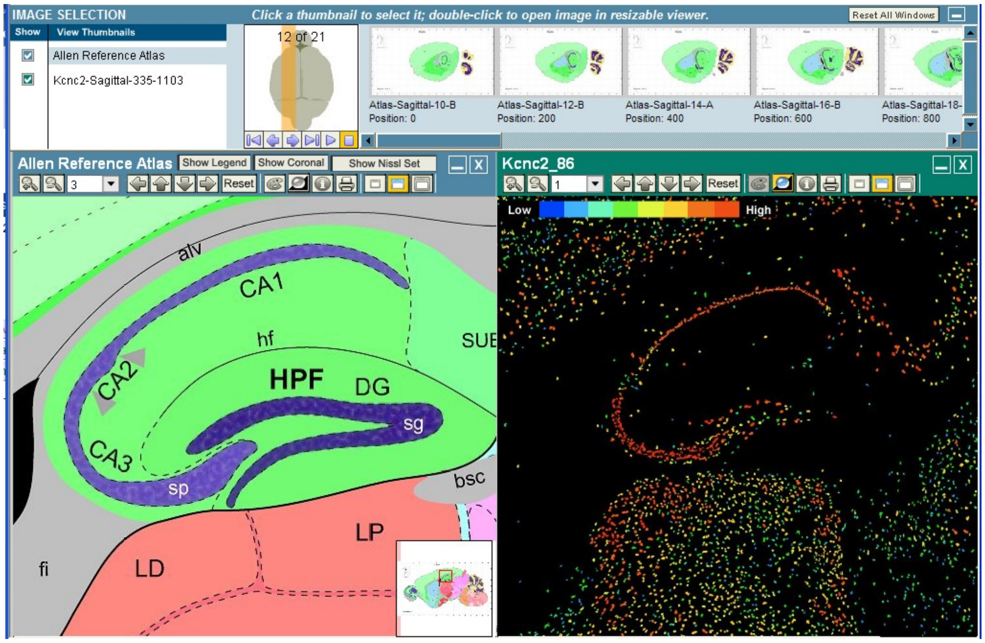

Sagital section of the Hippocampus shows strong Kv3.2 expression in CA3 neurons.

Supplemental Methods:

Preparation of Brain Slices

Brainstem slices containing the superior olivary complex (SOC) were prepared from CBA/Ca mice (Billups et al.), homozygous KN2 nNOS knockouts (Gyurko et al., 2002), Kv2.2 knockouts (B6;129S5-Kcnb2^{tm1Lex}; Lexicon Pharmaceuticals, 8800 Technology Forest Place, The Woodlands, TX 77381-1160, USA) or Kv3.1 knockouts (Macica et al., 2003) were obtained from colonies maintained at the University of Leicester. All KOs were back-crossed for 8 generation onto the CBA background.

All procedures were carried out according to the guidelines laid down by the University of Leicester animal welfare committee. Animals were killed by decapitation in accordance with the Animals, Scientific Procedures Act, 1986, and brain slices prepared as described previously (Wong et al., 2003) using animals aged between 13 and 16 days old. In brief, transverse slices of the SOC containing the MNTB were cut (200µm). Horizontal hippocampal slices of 250µm thickness were prepared as described previously (Brown and Randall, 2005) by mounting the whole brain onto a steel plate fitted onto a MicroSlicer DTK-1000.This procedure took place in low sodium artificial CSF (aCSF) at ~0°C, and slices were then stored at 37°C for 1h in normal aCSF, after which they were stored at room temperature (~20°C) until use. In a subset of experiments, brain slices were used immediately after slicing (earliest time point 10min). Composition of the normal aCSF was (mM): NaCl 125, KCl 2.5, $NaHCO_3$ 26, glucose 10, NaH_2PO_4 1.25, sodium pyruvate 2, myo-inositol 3, $CaCl_2$ 2, $MgCl_2$ 1 and ascorbic acid 0.5; pH was 7.4 when continuously bubbled with 95% O_2 / 5% CO_2. In the low sodium aCSF, NaCl was replaced by 250mM sucrose and $CaCl_2$ and $MgCl_2$ concentrations were 0.1 and 4mM, respectively.

Imaging and Electrophysiology

To detect synaptically connected MNTB neurons, slices were loaded with 5 µM Fura 2 AM (Molecular Probes, Eugene, OR, USA, dissolved in DMSO containing 5% pluronic acid) for 5-8 min in aCSF. Before recording, slices were kept in aCSF for 30 min to allow de-esterification of the AM dye. Fura 2 fluorescence was detected using imaging technique described previously (Billups et al., 2002) and viewed using a PentaMax intensified CCD camera (Princeton Instruments, Inc). The fluorescent image (emission >505nm) was displayed using Metafluor imaging software (version 7, Molecular Devices), the light source was a Polychrome II Monochromator (TILL Photonics, Martinsried, Germany). Midline stimulation evoked synaptic Ca^{2+} influx in principal MNTB neurons and whole cell recordings were made from those identified synaptically connected neurons, visualized with 60x objective on a Nikon E600FN microscope fitted with differential interference phase contrast optics. Patch recordings were

made using a Multiclamp 700B amplifier and pClamp 9.2 software (Molecular Devices), sampling at 50kHz and filtering at 10kHz. Patch pipettes were pulled from filamented borosilicate glass (GC150F-7.5, outer diameter 1.5 mm; inner diameter 0.86 mm; Harvard Apparatus, Edenbridge, UK) with a 2-stage vertical puller (PC-10 Narishige, Tokyo, Japan). Pipettes were used with final tip resistance of 2.5-3.5 MΩ when filled with a solution containing (mM): KCl 110, HEPES 40, EGTA 0.2, $MgCl_2$ 1, $CaCl_2$ 0.1, Na_2phosphocreatine 5, L-arginine 1; pH was adjusted to 7.2 with KOH. EPSCs in whole-cell configuration were elicited by stimulation through the bipolar electrode placed at the midline using the above parameters.

All recordings were taken at physiological temperature of 36±1°C, with the bath temperature being feedback controlled by a Peltier device warming aCSF passing through a fast-flow perfusion system, built by the University of Leicester Mechanical and Electronics workshop. A ceramic water-immersion objective coated with sylgard was used to minimize local heat loss. Single EPSCs were elicited at 0.2Hz to minimize desensitization and short term depression. Action potentials were elicited by injection of current steps (50pA). Drugs were obtained from Sigma UK, unless specified otherwise. Drugs were applied by bath perfusion in the aCSF.

Series resistances (R_S) for patch recordings were not exceeding 8MO and compensated by 70% and recordings with resistance changed over 20% were excluded from analysis. A voltage error associated with the clamping current passing across the series resistance will lead to a relative greater underestimation of larger currents; I/V curves were not corrected for R_s or leak subtracted.

Whole-cell patch recording gives exceptional resolution and voltage control but allows dialysis of the cytoplasm with the patch pipette solution. This can be advantageous in defining ionic composition and gradients, however it dilutes and attenuates intracellular signaling when control and experiment are measured from the same neuron (i.e. a test period follows a control interval but takes many minutes). Use of perforated patch (Korn and Horn, 1989) recording avoids dialysis but the high series resistances (>20 MΩ) compromise voltage clamp of large currents, so could not be employed here. EPSCs recorded in whole-cell configuration were elicited by stimulation of the trapezoid axons (in the brainstem) or the dentate region (including mossy fiber and commissural projections in the hippocampus) through a bipolar platinum electrode and isolated stimulator. Drugs and antagonists were applied by perfusion in the aCSF.

Input resistance (Rs) and membrane time constants (t) in CA3 pyramidal neurons (Rs: Ctrl: 140±27MO (n=12), NO: 95±14MO* (n=16), PC: 80±10MO* (n=8), P<0.05, ANOVA with post test; t: Ctrl: 34±6ms (n=12), NO: 23±4ms (n=16), PC: 18±2ms (n=8) and MNTB: (Rs: Ctrl: 88±15MO (n=37), NO: 78±18MO (n=12), PC: 90±9MO (n=10), P<0.05; t: Ctrl: 2.7±0.5ms (n=37), NO: 2.6±0.4ms (n=12), PC: 3.4±0.4ms (n=10). The extrapolated input resistance was determined

from an infinite time extrapolation of this fit. The extrapolated input resistance reflects resting input resistance prior to the additional activation of I_h that occurs upon hyperpolarization.

Fast time-course recordings following animal sacrifice

Brain slices containing the SOC were prepared as described above (no use of anesthetics) but immediately after slicing the tissue was moved into the preheated bath chamber and recordings were performed between 10 and 150min after sacrifice. It was not possible to conduct these fast recordings in hippocampal neurons as CA3 neurons could visually not be identified within 30min following slice procedures and only were able to patch-clamp after 30min incubation in aCSF.

Technical note: As dialysis during whole-cell patch recording rapidly blocks any NO signalling-induced modulation we avoided this by induction of the modulation prior to making the whole-cell recording (hence all recordings concerning nitrergic signalling are unpaired). The stereotypical properties of MNTB principal neurons and their compact electronic nature with few dendrites permitted good voltage-clamp and statistical validation using an unpaired method. An alternate strategy of using perforated patch methods was not practical because of high series resistances (20-50MΩ) inherent with this method which severely compromised clamp quality when passing large currents (i.e. a 5nA current passing across an uncompensated 10MΩ series resistance, will generate a 50mV command voltage error).

Immunohistochemistry

Brains were frozen in ‘Lamb OCT’ compound (Thermo Fisher Scientific) and cryostat sectioned at 12μm in the transverse plane. Sections were fixed in 4% paraformaldehyde at 4°C followed by antigen retrieval in 10mM citrate buffer (pH6) at 95^{0}C for 20min and subsequently incubated for 30min at room temperature with PBS containing 0.1% Triton X-100 (PBS-T), 1% BSA and 10% normal goat serum (NGS) to reduce non-specific binding of Abs. Sections were incubated with primary Abs to Kv3.1b (1:1000, NeuroMab), Kv3.3 (1:1000, Alomone), Kv3.4 (1:100, Alomone), Kv2.1 (1:100, Alomone), diluted in PBS-T containing 1% BSA and 10% NGS overnight at 4°C. After three washes in PBS-T, sections were incubated with secondary Abs (1:1000, Invitrogen; Molecular Probes anti-goat AlexaFluor 488 and 546 depending on primary Ab) diluted in PBS-T, 1% BSA and 10% NGS for 2h at room temperature. After rinsing in PBS-T, sections were stained with DAPI, rinsed again in PBS-T then cover-slipped with Vectashield Hard Set Mounting Medium (Vector Laboratories). Images were acquired with a Zeiss laser-scanning confocal

microscope (LSM 510, Carl Zeiss International). As negative controls for specificity, sections incubated with the omission of the primary Ab showed no specific immunostaining (data not shown) and pre-incubation with antigenic peptide (where available) also blocked specific staining. We have focused on Kv3.1b and Kv3.3 protein staining in the HC as we validated these two Abs in KO tissue. Kv3.2 Abs have not been used since we could not verify their specificity due to lack of KO mice. We have focused on Kv3.1b and Kv3.3 protein staining in the HC as we validated these two Abs in KO tissue. Kv3.2 Abs have not been used since we could not verify their specificity due to lack of KO mice.

Quantitative PCR

Tissue samples from the CA3 region of the hippocampus were excised from the same batch of frozen cryostat sections used for immunohistochemical staining using laser micro-dissection (PALM laser system, Zeiss). Total RNA extraction was performed using RNeasy tissue mini kit (Qiagen) and RNA was reverse-transcribed with SuperScript III (Invitrogen) using random hexamer primers (Promega). PCR primers were designed using the Primer Express version 2.0 Software program (Applied Biosystems, FosterCity, CA). Primers were designed to cross exon-exon regions and the gene of interest was normalised against a housekeeping gene (ß-actin). Primer sequences:

**Kv3.1a**	F- TCTGCAAAGCCTACGGATTC	R- AGGCTCAGCAAGGCTAAGG
**Kv3.1b**	F- CTTATCAACCGGGGAGTACG	R- AATGACAGGGCTTTCTTTGC
**Kv3.2**	F- GACCCGATGGCAAGTCAG	R- GAACAAAGAAGCAAAAGCAATAAA
**Kv3.3**	F- GACGTACCGCTCCACGTT	R- CCGGGTCGTAGTCAAAGC
**Kv3.4**	F- TCTTCGAGGATCCCTACTCATC	R- CGTTTCGGTCAATGTTGAAG
**ß-actin**	F- GATTACTGCTCTGGCTCCTAGCA	R- GTGGACAGTGAGGCCAGGAT

qPCR was performed using SYBR Green PCR Master Mix in the ABI PRISM 7700 Sequence Detection System, the thermal-cycler protocol was: stage one, 50°C for 2min; stage two, 95°C for 10 minutes; stage three, 40 cycles at 95°C for 15s and 60°C for 1min. Each sample was run in triplicate. PCR results were analysed using the Pfaffl model for relative quantification (Pfaffl, 2001).

Materials and drug application

All drugs were applied by bath perfusion in aCSF (AP-5 (Tocris, UK), MK-801 (Tocris, UK), 6-cyano-7-nitroquinoxaline-2,3-dione (CNQX, Tocris, UK), r-Stromatoxin-1 (Alomone), TEA (SIGMA), ODQ (Calbiochem), KT5823 (SIGMA), okadaic acid (SIGMA), SNP (Calbiochem), PapaNONOate (Cayman Chemical), 7-NI (SIGMA), GF109203X (SIGMA), Ro31-7549 (SIGMA), DCG-IV (Tocris, UK), iberiotoxin (Tocris, UK). NO donors (SNP and PapaNONOate) were made up fresh prior to the experiments and kept on ice and were light protected. Drugs were dissolved as stock solutions in water or DMSO, aliquoted and frozen at -20°C and diluted in aCSF immediately before the experiment.

References

Billups B, Wong AY, Forsythe ID (2002) Detecting synaptic connections in the medial nucleus of the trapezoid body using calcium imaging. Pflugers Arch 444:663-669.

Brown JT, Randall A (2005) Gabapentin fails to alter P/Q-type Ca^{2+} channel-mediated synaptic transmission in the hippocampus in vitro. Synapse 55:262-269.

Gyurko R, Leupen S, Huang PL (2002) Deletion of exon 6 of the neuronal nitric oxide synthase gene in mice results in hypogonadism and infertility. Endocrinology 143:2767-2774.

Johnston J, Griffin SJ, Baker C, Skrzypiec A, Chernova T, Forsythe ID (2008) Initial segment Kv2.2 channels mediate a slow delayed rectifier and maintain high frequency action potential firing in medial nucleus of the trapezoid body neurons. J Physiol 586:3493-3509.

Korn SJ, Horn R (1989) Influence of sodium-calcium exchange on calcium current rundown and the duration of calcium-dependent chloride currents in pituitary cells, studied with whole cell and perforated patch recording. J Gen Physiol 94:789-812.

Lein ES, Hawrylycz MJ, Ao N, Ayres M, Bensinger A, Bernard A, Boe AF, Boguski MS, Brockway KS, Byrnes EJ., et al (2007) Genome-wide atlas of gene expression in the adult mouse brain. Nature 445:168-176.

Macica CM, von Hehn CA, Wang LY, Ho CS, Yokoyama S, Joho RH, Kaczmarek LK (2003) Modulation of the Kv3.1b potassium channel isoform adjusts the fidelity of the firing pattern of auditory neurons. J Neurosci 23:1133-1141.

Pfaffl MW (2001) A new mathematical model for relative quantification in real-time RT-PCR. Nucleic Acids Res 29:e45.

Song P, Yang Y, Barnes-Davies M, Bhattacharjee A, Hamann M, Forsythe ID, Oliver DL, Kaczmarek LK (2005) Acoustic environment determines phosphorylation state of the Kv3.1 potassium channel in auditory neurons. Nat Neurosci 8:1335-1342.

Steinert JR, Kopp-Scheinpflug C, Baker C, Challiss RA, Mistry R, Haustein MD, Griffin SJ, Tong H, Graham BP, Forsythe ID (2008) Nitric oxide is a volume transmitter regulating postsynaptic excitability at a glutamatergic synapse. Neuron 60:642-656.

Wong AY, Graham BP, Billups B, Forsythe ID (2003) Distinguishing between presynaptic and postsynaptic mechanisms of short-term depression during action potential trains. J Neurosci 23:4868-4877.
